# Gut microbiota dependent anti-tumor immunity restricts melanoma growth in *Rnf5*^*−/*−^ mice

**DOI:** 10.1038/s41467-019-09525-y

**Published:** 2019-04-02

**Authors:** Yan Li, Roberto Tinoco, Lisa Elmén, Igor Segota, Yibo Xian, Yu Fujita, Avinash Sahu, Raphy Zarecki, Kerrie Marie, Yongmei Feng, Ali Khateb, Dennie T. Frederick, Shiri K. Ashkenazi, Hyungsoo Kim, Eva Guijarro Perez, Chi-Ping Day, Rafael S. Segura Muñoz, Robert Schmaltz, Shibu Yooseph, Miguel A. Tam, Tongwu Zhang, Emily Avitan-Hersh, Lihi Tzur, Shoshana Roizman, Ilanit Boyango, Gil Bar-Sela, Amir Orian, Randal J. Kaufman, Marcus Bosenberg, Colin R. Goding, Bas Baaten, Mitchell P. Levesque, Reinhard Dummer, Kevin Brown, Glenn Merlino, Eytan Ruppin, Keith Flaherty, Amanda Ramer-Tait, Tao Long, Scott N. Peterson, Linda M. Bradley, Ze’ev A. Ronai

**Affiliations:** 1 Sanford Burnham Prebys Medical Discovery Institute, La Jolla, CA 92037 USA; 2 Department of Food Science and Technology, University of Nebraska-Lincoln, Lincoln, NE 68588 USA; 3 Center for Bioinformatics and Computational Biology, Department of Computer Science, University of Maryland, College Park, MD 20742 USA; 4 School of Computer Sciences and Sackler School of Medicine, Tel Aviv University, Tel Aviv, Israel; 5 Laboratory of Cancer Biology and Genetics, Center for Cancer Research, National Cancer Institute, National Institutes of Health, Bethesda, MD 20892 USA; 6 Technion Integrated Cancer Center, Faculty of Medicine, Technion Israel Institute of Technology, Haifa, 31096 Israel; 7 Division of Medical Oncology, Massachusetts General Hospital Cancer Center, Harvard Medical School, Boston, MA 02114 USA; 8 Department of Computer Science, College of Engineering and Computer Science, University of Central Florida, Orlando, FL 32816 USA; 9 BioLegend, San Diego, CA 92121 USA; 10 Division of Cancer Epidemiology and Genetics, Laboratory of Translational Genomics, National Cancer Institute, Bethesda, MD 20892 USA; 11 Departments of Dermatology and Oncology, Rambam Health Care Campus, Technion Faculty of Medicine, Haifa, 31096 Israel; 12 Department of Pathology, Yale University, New Haven, CT USA; 13 Ludwig Institute for Cancer Research, Nuffield Department of Medicine, Old Road Campus, Unviversity of Oxford, Headington, Oxford, OX3 7DQ UK; 14 Department of Dermatology, University Hospital of Zurich and University of Zurich, 8091 Zurich, Switzerland; 15 Present Address: Department of Molecular Biology and Biochemistry, University of California, Irvine, Irvine, CA 92697 USA; 16 Present Address: Cancer Data Science Lab, National Cancer Institute, National Institutes of Health, Bethesda, MD 20892 USA

## Abstract

Accumulating evidence points to an important role for the gut microbiome in anti-tumor immunity. Here, we show that altered intestinal microbiota contributes to anti-tumor immunity, limiting tumor expansion. Mice lacking the ubiquitin ligase RNF5 exhibit attenuated activation of the unfolded protein response (UPR) components, which coincides with increased expression of inflammasome components, recruitment and activation of dendritic cells and reduced expression of antimicrobial peptides in intestinal epithelial cells. Reduced UPR expression is also seen in murine and human melanoma tumor specimens that responded to immune checkpoint therapy. Co-housing of *Rnf5*^−/−^ and WT mice abolishes the anti-tumor immunity and tumor inhibition phenotype, whereas transfer of 11 bacterial strains, including *B. rodentium*, enriched in *Rnf5*^−/−^ mice, establishes anti-tumor immunity and restricts melanoma growth in germ-free WT mice. Altered UPR signaling, exemplified in *Rnf5*^−/−^ mice, coincides with altered gut microbiota composition and anti-tumor immunity to control melanoma growth.

## Abstract (web summary)

RNF5 is a ubiquitin ligase regulating ER stress response. Here the authors show that Rnf5 deficiency potentiates immune response against melanoma via altered microbiota, and isolate bacterial strains that confer the same phenotype to wild type mice.

## Introduction

The clinical efficacy of immune checkpoint therapies has led to their use for a growing number of tumor types^1^. Despite this success, a significant proportion of patients exhibit resistance to immune checkpoint blockade^2^. As such a more detailed understanding of the molecular mechanisms underlying selectivity of the tumor response and control of immune checkpoint components is urgently needed. Unexpectedly, the efficiency of immune checkpoint therapy has been linked to the composition of the gut microbiota^3,4^. Although different studies have demonstrated that manipulation of the gut microbiota may impact antitumor immunity both in mice and human, the underlying molecular mechanisms are poorly understood. Notably, the identification of host genes/pathways that contribute to the regulation of the host microbiota and antitumor immunity is expected to be essential for the development of advanced therapeutic strategies.

RNF5 is a membrane-anchored E3 ubiquitin ligase implicated in endoplasmic reticulum (ER)-associated protein degradation (ERAD)^5^. RNF5 controls clearance of misfolded proteins, including mutant cystic fibrosis transmembrane regulator (CFTR)^6^ and glutamine carrier proteins, which are aberrantly folded following chemotherapy-induced ER stress (ERS)^7^. RNF5 also controls stability of the autophagy protein autophagy-related four homolog B (ATG4B)^8^. RNF5 was implicated in the control of stimulator of interferon genes (STING)^9^, a central signaling molecule in T-cell priming and immune checkpoint efficiency^10,11^. We thus investigated the potential effect of RNF5 on immune checkpoint control and tumor growth. Here, we demonstrate that *Rnf5*^−*/*−^ mice exhibits altered intestinal microbiota flora, which contributes to antitumor immunity, limiting tumor expansion.

## Results

### Antitumor immunity in *Rnf5*^*−/−*^ mice inhibits melanoma

**Fig. 1:**
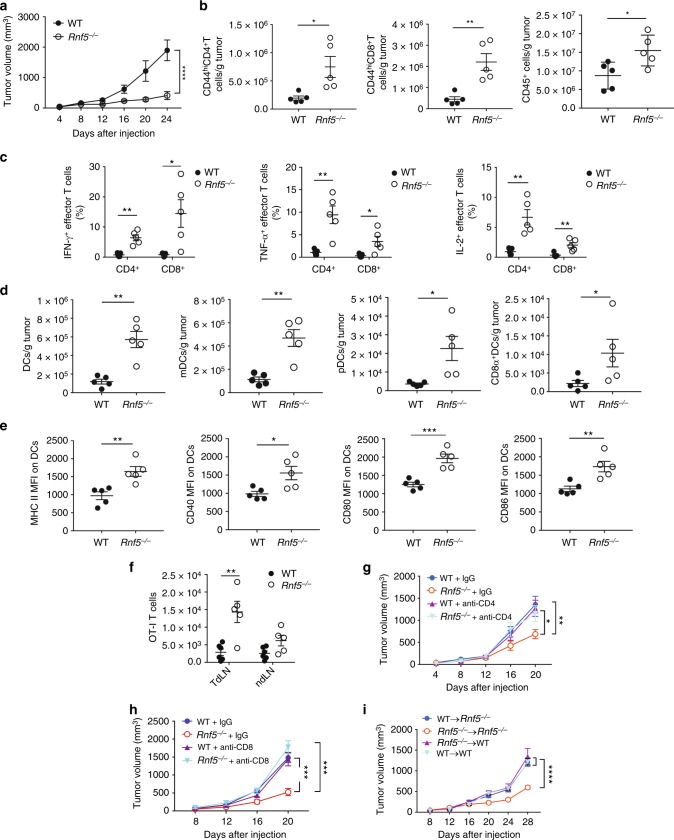
Enhanced antitumor immune responses in *Rnf5*^*−/−*^ mice. **a** Growth of YUMM1.5 (*Braf*^*V600E*^*:PTEN*^*−/−*^*:Cdkn2a*^*−/−*^) melanoma cells after subcutaneous injection of 10^6^ cells into WT or *Rnf5*^*−/−*^ mice (*n* = 5). **b** Quantification of tumor-infiltrating effector (CD44^hi^) CD4^+^ and CD8^+^ T cells and total CD45^+^ cells on day 24 after tumor injection (*n* = 5). **c** Frequencies of tumor-infiltrating TNF-α-, IFN-γ-, and IL-2-producing CD4^+^ and CD8^+^ T cells on day 24 after tumor inoculation (*n* = 5). **d** Quantification of tumor-infiltrating total DCs, pDCs, mDCs, and CD8α^+^ DCs on day 24 after tumor inoculation (*n* = 5). **e** Expression (mean fluorescence intensity, MFI) of MHC class II, CD40, CD80, and CD86 on tumor-infiltrating DCs (CD45^+^ CD11c^+^) on day 24 after tumor inoculation (*n* = 5). **f** Quantification of OT-I CD8^+^ T cells in tumor-draining lymph nodes (TdLN) and non-draining lymph nodes (ndLN) of CD45.1^+^ WT and *Rnf5*^*−/−*^ mice injected with B16-OVA melanoma cells (WT, *n* = 6; *Rnf5*^*−/−*^, *n* = 5). **g**, **h** Growth of YUMM1.5 melanoma cells in mice injected i.p. with control IgG and anti-CD4 (**g**) or control IgG and anti-CD8 (**h**) depleting antibodies on days 0, 3, 6, 11, 16 (*n* = 9). FACS analysis revealed >90% depletion of blood CD4^+^ and CD8^+^ T cells on day 7 after tumor inoculation. **i**, Growth of YUMM1.5 melanoma cells in lethally irradiated bone marrow-reconstituted WT or *Rnf5*^*−/−*^ mice (arrow indicates bone marrow donor → recipient; *n* = 7). Data are representative of three independent experiments (**a**–**e**), two independent experiments (**f**, **i**) and one experiment (**g**, **h**) ≥5 mice per group. Graphs show the mean ± s.e.m. *P < 0.05, **P < 0.005, ***P < 0.001, ****P < 0.0001 by two-way ANOVA with Sidak’s correction (**a**, **g**–**i**) or by two-tailed *t-*test or Mann–Whitney *U* test (**b**–**f**)

To determine whether *Rnf5*^*−/−*^ mice exhibit altered antitumor immune response, we evaluated the growth of a series of mouse melanoma cell lines injected subcutaneously into the flank of syngeneic *Rnf5*^−/−^ C57BL/6 mice obtained by crossing of *Rnf5* heterozygotes. Tumors arising from YUMM1.3, YUMM1.5, and YUMM1.9 cells (all *Braf*^*V600E*^*:Pten*^−/−^*:Cdkn2a*^−/−^)^12^, or B16F10 melanoma cells, or from shRNF5-expressing YUMM1.3 cells, all grew to the same extent over the first week followed by slower growth resulting in significantly smaller tumors in *Rnf5*^*−/−*^ compared with wild-type (WT) mice (Fig. 1a; Supplementary Figure 1A). Correspondingly, tumor-bearing *Rnf5*^*−/−*^ mice exhibited better survival, compared with the WT genotype (Supplementary Figure 1B). These data raised the possibility that RNF5 within the host would contribute to the control of antitumor immunity. The antitumor immune response was interrogated by using fluorescence-activated cell sorting (FACS) analysis of tumor-infiltrating cells isolated on days 16 and 24 after tumor cell injection. The results showed a marked enrichment of total CD45^+^ cells and effector (CD44^hi^) CD8^+^ and CD4^+^ T cells in tumors from *Rnf5*^−/−^ versus WT mice (Fig. 1b; Supplementary Figure 1C). *Rnf5*^−/−^ tumor-infiltrating CD4^+^ and CD8^+^ lymphocytes (TILs) also displayed greater effector function, including enhanced IFN-γ, TNF-α, and IL-2 production (Fig. 1c; Supplementary Figure 1D). These data suggest that superior recruitment and effector function of TILs might underlie the more potent antitumor response observed in *Rnf5*^−/−^ mice. In support of this, the total number of dendritic cells (DCs), including myeloid (mDCs), plasmacytoid (pDCs), and CD8^+^ conventional DCs, were higher in tumors from *Rnf5*^−/−^ than WT mice (Fig. 1d). *Rnf5*^−/−^ DCs also expressed higher levels of MHC class II and the costimulatory molecules CD40, CD80, and CD86 (Fig. 1e). Expression of MHC class II as well as CD80 and CD86 molecules was also higher on tumor-infiltrating macrophages from *Rnf5*^−/−^ compared with WT mice (Supplementary Figure 1E). Of note, expression of the inhibitory checkpoint receptors PD-1, T-cell immunoglobulin and mucin domain 3 (TIM-3), and lymphocyte-activation 3 (LAG-3) were also upregulated on *Rnf5*^−/−^ CD8^+^ T cells (Supplementary Figure 1F) and PD-L1 expression was upregulated on *Rnf5*^−/−^ macrophages and DCs (Supplementary Figure 1G), further implying that enhanced immune stimulation in *Rnf5*^−/−^ mice overcomes immune checkpoint-mediated inhibition of antitumor response. Altogether, these data indicate a clear shift to a proinflammatory tumor microenvironment in *Rnf5*^−/−^ mice.

To provide independent support for a role for tumor-specific T cells in the antitumor response of *Rnf5*^−/−^ mice, we transferred OVA-specific OT-I transgenic CD8^+^ T cells into WT or *Rnf5*^−/−^ recipient mice and then injected recipients subcutaneously with OVA-expressing B16F10 melanoma cells. Analysis of tumor-draining and non-draining lymph nodes revealed that OT-I CD8^+^ T cells were more abundant in the draining lymph nodes of *Rnf5*^−/−^ compared with WT mice (Fig. 1f; Supplementary Figure 1H). Since the degree of T-cell proliferation was comparable in both genotypes (Supplementary Figure 1I), we attribute their greater abundance to their improved survival or greater recruitment of OT-1 cells into the draining LN in *Rnf5*^*−/−*^ microenvironments. Since OT-1 cells are engaged in early priming events, their analysis is restricted to lymph nodes and not tumors, which were collected at later times. These results indicate that the improved immune response observed in *Rnf5*^*−/−*^ mice occurs upstream of T-cell expansion, likely at the level of host DCs.

The importance of the immune system for tumor control seen in the *Rnf5*^−/−^ mice was confirmed upon administration of antibodies depleting CD4^+^ (Fig. 1g) or CD8^+^ (Fig. 1h) T cells, which abrogated the ability of *Rnf5*^−/−^ mice to inhibit melanoma tumor growth, with a small additive effect upon CTLA4, but not PD-1 blockade (Supplementary Figure 1J, K). These results point to a critical role for RNF5 in the CD4^+^ and CD8^+^ T cell-dependent antitumor immune response.

We asked next whether the observed antitumor immunity in *Rnf5*^−/−^ mice was due to loss of RNF5 in hematopoietic or non-hematopoietic cells. To do so, we examined tumor growth in the bone marrow (BM) chimeric mice created by injection of either WT or *Rnf5*^−/−^ bone marrow cells into lethally irradiated WT or *Rnf5*^−/−^ recipients. While tumor growth in *Rnf5*^−/−^ → *Rnf5*^−/−^ chimeras was inhibited, this was not the case following BM transfer from WT → *Rnf5*^−/−^ and *Rnf5*^*−/−*^ → WT chimeras, where tumor growth was comparable with that in WT BM → WT mice (Fig. 1i). These observations provided the first indication that a non-hematopoietic compartment is also required for the antitumor response seen in the *Rnf5*^−/−^ mice.

### TLRs are required for antitumor response in *Rnf5*^*−/−*^ mice

**Fig. 2:**
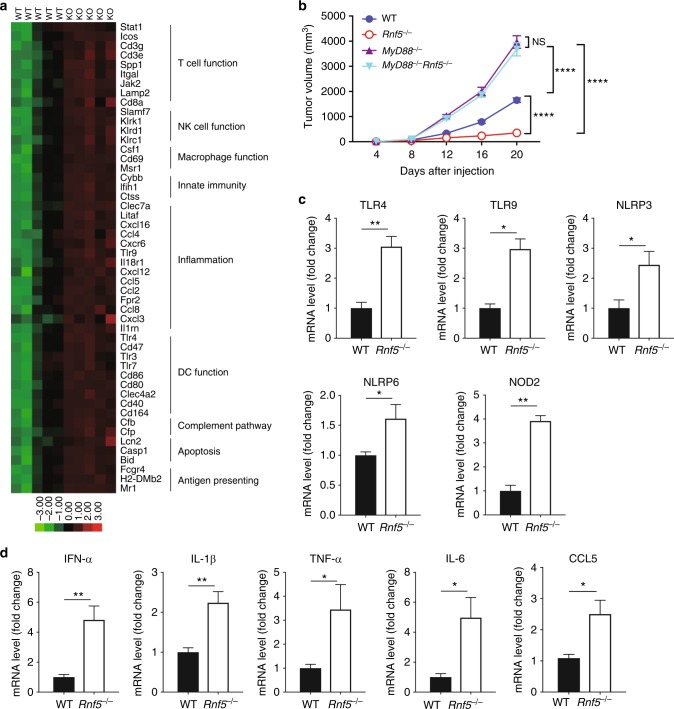
Enhanced inflammasome and pathogen receptor signaling in *Rnf5*^*−/−*^ mice. **a** NanoString analysis of PanCancer Immune Profiling genes in tumors from WT and *Rnf5*^*−/−*^ mice. The heatmap shows 47 genes with >1.2-fold (*P* < 0.05) differences in expression between YUMM1.5 tumors from the two genotypes (*n* = 5) at 10 days after injection. **b** Growth of B16F10 melanoma cells after subcutaneous injection of 10^6^ cells into WT, *Rnf5*^*−/−*^, *MyD88*^−*/−*^ and *MyD88*^*−/*−^*Rnf5*^*−/*−^ mice (WT, *Rnf5*^*−/−*^, *n* = 6; *MyD88*^*−/−*^, *n* = 5; *MyD88*^*−/−*^ *Rnf5*^*−/−*^, *n* = 8). **c** qRT-PCR analysis of pathogen receptor mRNA levels in IECs from tumor-bearing WT or *Rnf5*^*−/−*^ mice (*n* = 4). **d** qRT-PCR analysis of cytokine and chemokine mRNA levels in IECs isolated from the small intestines of tumor-bearing WT or *Rnf5*^*−/−*^ mice (*n* = 4). Data are representative of three independent experiments (**c**, **d**), two independent experiments (**b**) or one experiment (**a**) ≥5 mice per group. Graphs show the mean ± s.e.m. *P < 0.05, **P < 0.005, ***P < 0.001, ****P < 0.0001 by two-tailed *t* test or Mann–Whitney *U* test (**c**, **d**) or two-way ANOVA with Sidak’s correction (**b**)

To identify potential differences in immunoregulatory gene expression in WT and *Rnf5*^−/−^ mice, we performed NanoString analysis of 770 genes expressed by 24 immune cell types in the tumors at 10 days after injection. This analysis uncovered marked differences in key immune regulatory networks associated with T, DC, natural killer (NK), and macrophage cell function (Fig. 2a; Supplementary Figure 2A). Interestingly, changes in expression of chemokines and genes related to innate immunity, antigen presentation, and DC functional networks suggested a possible role for TLRs in *Rnf5*^−/−^ mouse phenotypes (Supplementary Figure 2B). Changes in gene expression identified by NanoString analysis were confirmed by qPCR analysis of cDNA derived from tumors grown in WT and *Rnf5*^*−/−*^ mice (Supplementary Figure 2C). Chemokine (C–C motif) ligand 5 (CCL5), which is associated with TLR signaling, was also upregulated in serum from tumor-bearing *Rnf5*^−/−^ compared with WT mice (Supplementary Figure 2D). To this end, we generated mice lacking both myeloid differentiation primary response 88 (MyD88), a key adaptor downstream from TLR signaling, and RNF5 (*MyD88*^*−/−*^*:Rnf5*^−/−^). Importantly, melanoma growth was no longer inhibited in *MyD88*^*−/*−^*Rnf5*^−/−^ mice, with *MyD88*^*−/−*^ mice exhibiting a tumor growth phenotype between that of the WT and *MyD88*^−/−^*Rnf5*^−/−^ double-knockout mice (Fig. 2b). These data demonstrate a role for the TLRs including their master regulator MyD88 in the enhanced antitumor immunity induced in *Rnf5*^*−/−*^ mice, while the role of IL-IR can not be excluded.

Our results suggested that the enhanced antitumor immune response in *Rnf5*^*−/−*^ mice involved both TLRs signaling as well as a non-hematopoietic component. We therefore assessed possible changes in both TLRs and inflammasome components of intestinal epithelial cells (IECs), which have been previously associated with an altered gut microbiota composition and enhanced antitumor immunity^13–15^. Indeed, expression of TLR4 and TLR9 and also that of pathogen-associated molecular pattern receptor signaling pathways and the inflammasome components nucleotide-binding oligomerization domain 2 (NOD2), NLR family pyrin domain containing 3 (NLRP3), and NOD-like receptor family pyrin domain 6 (NLRP6) was upregulated in IECs from tumor-bearing *Rnf5*^−/−^ mice (Fig. 2c), not naive *Rnf5*^−/−^ mice (Supplementary Figure 2E). Enhanced cytokine and chemokine expression were observed in IECs from tumor-bearing *Rnf5*^−/−^ mice (Fig. 2d). These activities likely underlie TLR activation and the concomitant antitumor phenotypes seen in *Rnf5*^−/−^ mice.

### Gut microbiota defines antitumor immunity in *Rnf5*^*−/−*^ mice

**Fig. 3:**
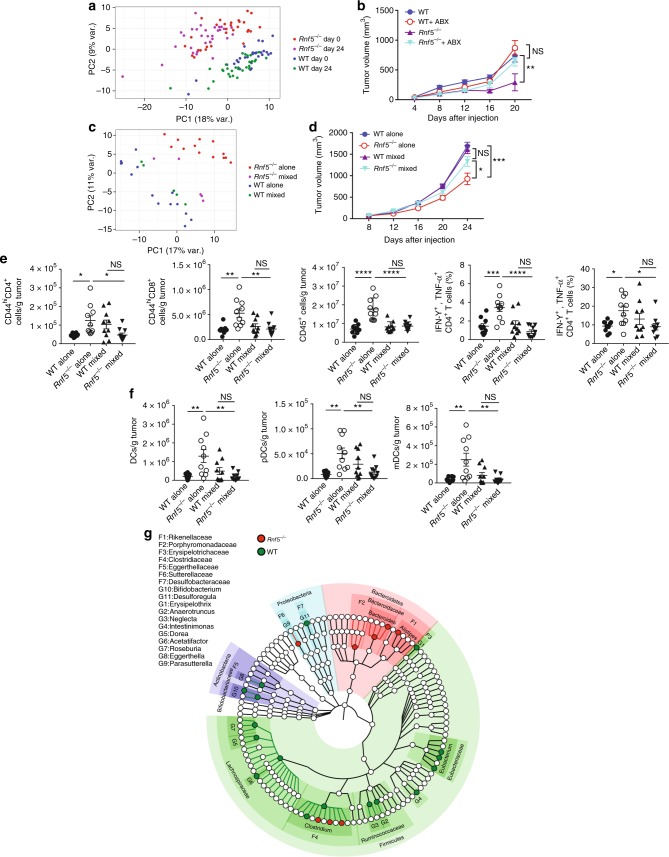
Gut microbiota control melanoma growth. **a** Principal component analysis (PCA) of all taxa enumerated in *Rnf5*^−/−^ and WT fecal microbiota samples taken before (day 0) and 24 days after injection of YUMM1.5 tumor cells (*n* = 30). **b** Elimination of tumor growth suppression in *Rnf5*^*−/−*^ mice by treatment with an antibiotic cocktail administered for 2 weeks prior to tumor cell injection (*n* = 5). **c** PCA of all taxa showing convergence of gut microbiota in WT and *Rnf5*^*−/−*^ mice after co-housing (WT alone, *n* = 14; *Rnf5*^*−/*−^ alone, *n* = 15; WT mixed, *Rnf5*^−*/−*^ mixed, *n* = 5). **d** Growth of YUMM1.5 melanoma cells in WT or *Rnf5*^−/−^ mice housed alone or co-housed (mixed) for 4 weeks prior to tumor inoculation (alone, *n* = 15; mixed, *n* = 16). **e** Quantification of effector (CD44^hi^) CD4^+^ and CD8^+^ T cells, total CD45^+^ cells, and frequencies of IFN-γ + TNF-α-producing CD4^+^ and CD8^+^ T cells in tumors from WT or *Rnf5*^−/−^ mice housed alone or co-housed for 4 weeks prior to tumor inoculation (*n* = 10). **f** Quantification of tumor-infiltrating total DCs, pDCs, and mDCs in WT or *Rnf5*^−/−^ mice housed alone or co-housed for 4 weeks prior to tumor inoculation (*n* = 10). **g** A cladogram representation of taxa enriched in *Rnf5*^*−/−*^ mice (red) microbiota and taxa enriched in WT mice (green) microbiota (*n* = 30). The background color indicates phylum; names of phyla are indicated. Data are representative of two independent experiments ≥5 mice per group. Graphs show the mean ± s.e.m. *P < 0.05, **P < 0.005, ***P < 0.001, ****P < 0.0001 by one-way ANOVA with Tukey’s (**e**, **f**) or two way ANOVA with Sidak’s (**b**, **d**) correction for multiple comparisons

Growing evidence supports the importance of the gut microbiome in control of immune surveillance and tumor responses to therapy^3,4^. We therefore analyzed whether phenotypes seen in *Rnf5*^−/−^ mice were linked to, or affected by, changes in composition of the gut microbiota. To map the composition of the gut microbiota in both WT and *Rnf5*^−/−^ mice, we performed 16S sequencing of V3–V4 regions followed by computational analysis. Principal component analysis revealed marked differences in microbial community structure (Fig. 3a), differences that distinctly segregated *Rnf5*^*−/−*^ from WT microbiota. We therefore asked whether differences in gut microbiota composition might underlie the phenotypes of tumor growth inhibition and enhanced antitumor immunity seen in the *Rnf5*^−/−^ mice. Two key experiments were performed to address this question. First, mice received an antibiotic cocktail (ABX) in drinking water 2 weeks before tumor inoculation. Strikingly, ABX treatment largely prevented the inhibition of tumor growth inhibition seen in *Rnf5*^−/−^ mice (Fig. 3b), suggesting that the gut microbiota play a key role in the control of tumor growth in the *Rnf5*^−/−^ mice. Second, co-housing of mice, that is known to compromise such differences between mice harboring distinct microbiota commensals^16^, notably diminished segregation of the bacterial landscapes seen when genotypes were housed separately (Fig. 3c). Melanoma growth in *Rnf5*^−/−^ mice that were co-housed with WT mice, was no longer inhibited (Fig. 3d), again pointing to the importance of gut microbiota components present in *Rnf5*^−/−^ for limiting tumor growth. Notably, loss of antitumor immunity seen in the *Rnf5*^−/−^ mice coincided with the loss of tumor growth inhibition following co-housing. Thus, co-housing of the *Rnf5*^−/−^ with the WT mice led to reduced numbers of TILs, including CD44^hi^ CD4^+^, CD44^hi^ CD8^+^ T cells, and CD45^+^ cells, concomitant with decreased frequencies of cytokine-producing T cells (Fig. 3e), decreased numbers of DCs and DC subsets (Fig. 3f), and lower MHC class II expression on DCs (Supplementary Figure 3A). These observations establish the importance of the gut microbiota in *Rnf5*^−/−^ mice in the control of antitumor immunity and tumor growth inhibition.

### Bacterial strains are enriched in the gut microbiota of *Rnf5*^*−/−*^ mice

Sequencing of the amplified 16S V3–V4 region followed by computational analyses, led to the identification of 38 taxa that distinguished the microbiomes of tumor-bearing *Rnf5*^−/−^ from tumor-bearing WT mice (Fig. 3g; Supplementary Table 1). The cooccurrence of these taxa is notable as inter-correlated clusters are formed based on correlation of abundance (Supplementary Figure 3B). These taxa were dominated by those positively correlated with tumor size, whereas only 11 taxa displayed a significant negative correlation with tumor size (Supplementary Figure 3C, D). Positively correlated taxa were highly enriched in members of the Firmicutes phylum, particularly members of Clostridiales, whereas negatively correlated taxa were enriched for members of Bacteroides and Parabacteroides.

Since differentially abundant phylotypes exist in the background of strains and species belonging to target genera that may or may not be altered in *Rnf5*^−*/*−^ mice, we assessed the absolute abundance of Bacteroides, Alistipes, Lactobacillus, and Bacteroides massiliensis. No difference was found in absolute bacterial abundance of Bacteroides or Alistipes comparing WT and *Rnf5*^*−/−*^ mice in either naive or tumor-bearing mice. A decrease in the absolute abundance of Lactobacillus in tumor-bearing *Rnf5*^−*/−*^ mice was found, compared with tumor-bearing WT mice, and an increase in Bacteroides massiliensis was identified in naive *Rnf5*^−*/*−^ mice, compared with naive WT mice (Supplementary Figure 3E).

### Bacterial strains confer antitumor immunity in GF WT mice

**Fig. 4:**
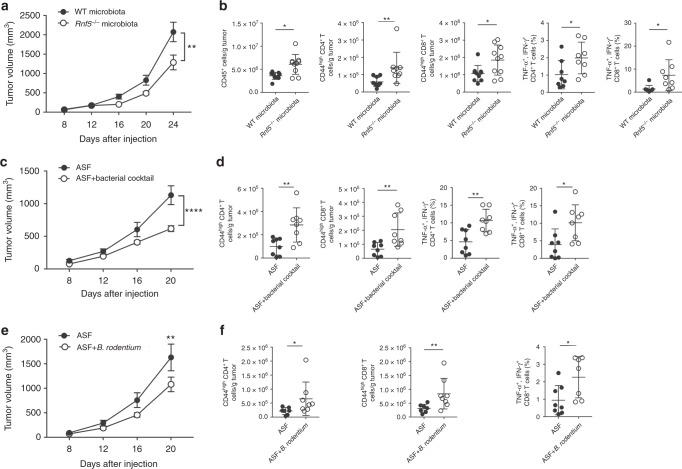
Oral administration of select bacterial strains enriched in *Rnf5*^*−/−*^ mice to gnotobiotic mice enhances antitumor immune response. **a** YUMM1.5 tumor growth in germ-free (GF) mice undergoing oral gavage with WT or *Rnf5*^*−/−*^ cecal contents 2 weeks prior to tumor implantation (*n* = 10). **b** Quantification of tumor-infiltrating CD45^+^ cells, effector (CD44^hi^) CD4^+^ and CD8^+^ T cells, total CD45^+^ cells, and frequencies of IFN-γ^+^TNF-α^+^-producing CD4^+^ and CD8^+^ T cells on day 24 after tumor injection in recipients treated as in (**a**) (*n* = 10). **c** YUMM1.5 tumor growth in GF mice undergoing oral gavage with either Altered Schaedler Flora (ASF) or ASF plus bacterial cocktail prior to tumor inoculation (ASF, *n* = 12; ASF + bacterial cocktail, *n* = 14). **d** Quantification of tumor-infiltrating effector (CD44^hi^) CD4^+^ and CD8^+^ T cells and frequencies of IFN-γ^+^TNF-α^+^-producing CD4^+^ and CD8^+^ T cells on day 21 after tumor injection in mice treated as in (**c**) (*n* = 8). **e** YUMM1.5 tumor growth in GF mice undergoing oral gavage with ASF or ASF plus *B. rodentium* prior to tumor inoculation (*n* = 15). **f** Quantification of tumor-infiltrating effector (CD44^hi^) CD4^+^ and CD8^+^ T cells and frequencies of IFN-γ^+^TNF-α^+^-producing CD8^+^ T cells on day 24 after tumor injection in mice treated as in (**e**) (*n* = 8). Data are one experiment ≥8 mice per group.Graphs are the mean ± s.e.m. *P < 0.05, **P < 0.005, ****P < 0.0001 by two-way ANOVA with Tukey’s or Sidak’s correction for multiple comparisons (**a**, **c**, **e**) or two-tailed *t* test or Mann–Whitney *U* test (**b**, **d**, **f**)

To directly test whether the gut microbiota regulated antitumor immunity, we transferred cecal contents from either WT or *Rnf5*^*−/−*^ into germ-free (GF) mice via oral gavage 2 weeks prior to tumor implantation. Prophylactic transfer of *Rnf5*^*−/−*^ microbiota was sufficient to delay tumor growth (Fig. 4a), as well as to enhance infiltration of tumor-specific CD45^+^, CD4^+^, CD8^+^ T cells and increase cytokine production (Fig. 4b), supporting a role for the *Rnf5*^*−/−*^ microbiota in mediating antitumor effects.

We next created a cocktail of 12 bacterial strains that displayed a significant negative correlation with tumor size and were overrepresented in *Rnf5*^*−/−*^ mice compared with WT counterparts (Supplementary Table 2). GF recipient mice were then administered either Altered Schadler Flora (ASF) or ASF plus that cocktail. Administration of the latter to GF mice markedly inhibited melanoma growth (Fig. 4c), which was associated with an increased infiltration of T cells and an enhanced antitumor cytokine response (Fig. 4d) compared with GF mice administered ASF alone. Likewise, we have examined whether the select bacterial strains were also capable of eliciting antitumor immunity to NRAS mutant melanoma (SW1 cells), using a different mouse strain (C3H/HeN). Administration of bacterial cocktail into ASF co-colonized mice attenuated growth of SW1 tumors (Supplementary Figure 4A), indicating that the effect of this bacterial cocktail is not limited to C57BL/6 GF mice, and is also effective in different genetic mouse strains and melanomas of diverse mutational backgrounds. Notably, one of the 12 strains of the cocktail was *B. rodentium*, which was also enriched in animals that underwent anti-CTLA-4 therapy (Supplementary Figure 4B). Administration of *B. rodentium* to gnotobiotic mice receiving ASF mildly, but statistically significantly, attenuated tumor growth relative to mice treated with ASF alone (Fig. 4e). Notably, tumor growth inhibition seen following administration of *B. rodentium* was also accompanied by enhanced tumor infiltration of CD4^+^ and CD8^+^ T cells and cytokine production (Fig. 4f), confirming activation of antitumor immunity by specific gut microbiota. Overall, these findings establish the critical role of the select gut microbiota commensals in the activation of antitumor immunity, resulting in tumor growth inhibition. Interestingly, co-culture of mouse duodenal epithelial cells, MODE-K cells, with *B. rodentium* also induced activated components of the TLR signaling pathway and ER stress signature (Supplementary Figure 4C), pointing to a feed forward loop mechanism where IEC not only activate inflammasomes with concomitant impact on TLR, but can be also affected by select bacterial strains to turn on UPR or other forms of stress (i.e., oxidative stress^17–19^).

### Decreased antimicrobial peptides in *Rnf5*^*−/−*^ mice

**Fig. 5:**
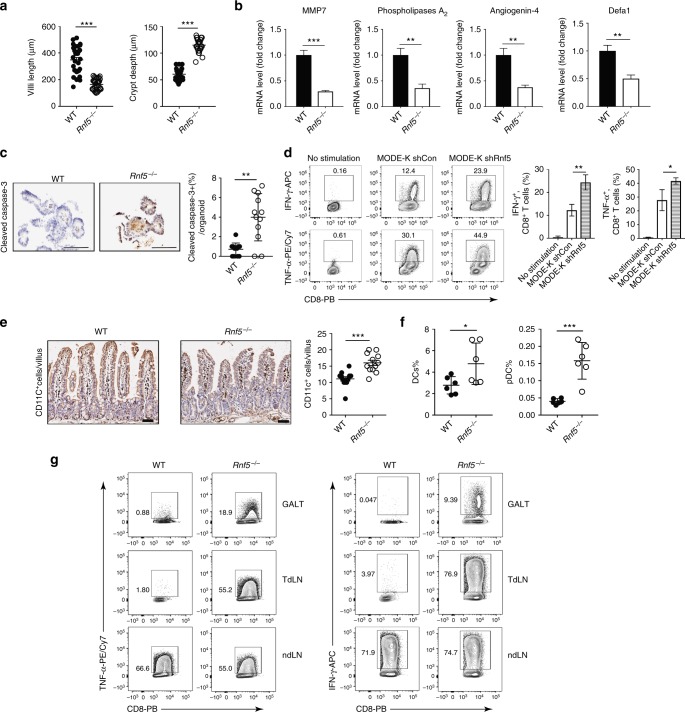
IEC of *Rnf5*^*−/−*^ mice activate immune response and change anti-microbial peptide (AMP) expression. **a** Villi length and crypt depth calculated from H&E-stained sections of intestines from WT or *Rnf5*^−/−^ mice (WT, *n* = 30; *Rnf5*^*−/−*^, *n* = 32). **b** qRT-PCR analysis of AMPs mRNA levels in IECs from small intestine of naive WT or *Rnf5*^*−/−*^ mice (*n* = 6). **c** Representative images (left) and quantification (right) of cleaved caspase-3 immunostained small intestine organoids from tumor-bearing WT or *Rnf5*^*−/−*^ mice (*n* = 3). Scale bar = 100μm. Graph shows percentage of cleaved caspase-3 ^+^ cells per immunostained organoid (*n* = 12 fields). **d** Intracellular IFN-γ and TNF-α staining of p14 CD8^+^ T cells incubated for 72 h with 2 μg/ml GP33 peptide recognized by the TCR of P14 and bone marrow-derived dendritic cells (BMDCs) that were incubated with medium alone (no stimulation) or with conditioned medium (CM) from shControl or shRNF5 MODE-K cells. **e** Representative images (left) and quantification (right) of CD11c^+^ cell immunostaining in the small intestine of WT or *Rnf5*^*−/−*^ mice on day 24 after injection of YUMM1.5 cells. Scale bar = 50μm (*n* = 4). **f** Frequencies of total DCs and pDCs in Peyer’s patches from WT and *Rnf5*^*−/−*^ mice on day 10 after YUMM1.5 cell injection (*n* = 6). **g** 10 days after tumor injection, DCs from GALT, dLN, and ndLN were isolated, pooled per group (*n* = 10 mice/group), and were incubated with OT-1 CD8^+^ T cells stimulated with 2 μg/ml OVA peptide (SINFEKL). Intracellular IFN-γ and TNF-α of OT-1 CD8^+^ T cells were detected. Data are representative of three independent experiments (**b**, **d**) and two independent experiments (**a**, **c**, **e**, **f**, **g**) ≥3 mice per group. Graphs show the mean ± s.e.m. *P < 0.05, **P < 0.005, ***P < 0.001, ****P < 0.0001 by two-tailed *t* test or Mann–Whitney *U* test (**a**, **b**, **c**, **e**, **f**) or one-way ANOVA with Tukey’s (**d**) correction for multiple comparisons

We characterized the inflammatory status of serum and gut epithelial barrier in *Rnf5*^*−/−*^ mice. Elevated serum cytokines were observed in naive *Rnf5*^*−/−*^ mice (Supplementary Figure 5A). Shortening of villi and increasing crypt depth were observed in the small intestine in naive *Rnf5*^*−/−*^ mice (Fig. 5a; Supplementary Figure 5B). Notably, co-housing with WT mice abolished villi length and crypt depth differences, implicating gut microbiota commensals in control of innate immune phenotypes (Supplementary Figure 5C). Furthermore, the expression pattern of a number of antimicrobial peptides (AMPs), implicated in the regulation of gut microbiota composition, was markedly reduced in the intestine of *Rnf5*^−/−^ mice (Fig. 5b), Moreover, we observed increased cell death in organoids prepared from the IEC of tumor-bearing *Rnf5*^−/−^ mice (Fig. 5c). These data suggest decreased AMPs and increased cell death might cause intestinal dysbiosis in *Rnf5*^−/−^ mice.

### RNF5 loss in IEC activates immune cells by CCL5 production

We next assessed whether IECs from *Rnf5*^−/−^ mice exhibit a better capacity to activate immune cells. *Rnf5*-depleted MODE-K cells, a mouse intestinal epithelial line also exhibits enhanced chemokine CCL5 expression (Supplementary Figure 5D). Significantly, co-culture of supernatants from cultures of RNF5-depleted MODE-K cells with murine bone marrow-derived DCs (BMDCs) enhanced DC activation, seen by elevated MHC I and MHC II expression (Supplementary Figure 5E). This finding is consistent with reports identifying the activation of immature DC by chemokines^20^. Furthermore, the addition of supernatants from cultures of RNF5-depleted MODE-K cells to co-cultures of BMDCs and T-cell receptor (TCR) transgenic T cells with their TCR-specific peptide led to increased T-cell response, as measured by increased cytokine production (Fig. 5d). Together, these observations suggest that altered signaling in IEC lacking RNF5 stimulates DCs and consequently T cells.

### Enhanced activity of DCs in the gut of *Rnf5*^*−/−*^ mice

We next asked whether alterations in IECs derived from *Rnf5*^−/−^ mice changed immune cell recruitment and activity. We observed a significant increase in CD11c^+^ DCs in the small intestine of *Rnf5*^−/−^ versus WT mice (Fig. 5e), consistent with previous reports implicating CD11c^+^ DCs in triggering enhanced antitumor immune response^21,22^. Of equal importance, we observed that both DCs and pDCs were significantly more abundant in Peyer’s patches (PP) from tumor-bearing *Rnf5*^*−/−*^ mice (Fig. 5f) than in naive animals (Supplementary Figure 5F). Furthermore, compared with DCs isolated from the PPs of the WT genotype, DCs of *Rnf5*^*−/−*^ mice produced higher levels of IL-1β in response to TLR7 stimulation, higher levels of IL-1β, IL-17A, and IL-27 in response to TLR9 stimulation, and lower levels of IL-10 in response to both TLR7 and TLR9 stimulation (Supplementary Figure 5G). Additionally, increased production of CCL5, CCL22, CXCL1, and CXCL5 chemokines was found in *Rnf5*^*−/−*^ PPs DCs after TLR7 stimulation (Supplementary Figure 5H). The response of PPs-derived DCs to TLR7 and TLR9 agonists is consistent with their tissue-specific activity^23^. These data suggest that DCs from Peyer’s patches of *Rnf5*^*−/−*^ mice better promote the immune response. Notably, DCs isolated from the gut-associated lymphoid tissue (GALT) including PP, mesenteric lymph node (MLN), and tumor-draining lymph node (TdLN) of tumor-bearing *Rnf5*^*−/−*^ mice induced more T cell cytokine production compared with DCs isolated from WT tumor-bearing mice (Fig. 5g). Intestinal dendritic cells were shown to upregulate CCR7 before migrating out of the intestine to lymphoid tissues under inflammatory conditions^24,25^. Likewise, tumor-bearing *Rnf5*^*−/−*^ mice had enhanced frequency of CCR7^+^ DCs isolated from GALT and of CCR7^+^ mDCs isolated from MLN, compared with those isolated from the WT tumor-bearing mice (Supplementary Figure 5I). This data suggest that a higher fraction of migratory intestinal DCs are present in tumor-bearing *Rnf5*^*−/−*^ mice. In addition, CD103^+^ DCs are implicated in the transport of intact antigens to the TdLN, thus priming CD8^+^ T cells^26^. An increase in CD103^+^ DCs was observed in TdLN, but not in non-draining lymph node (ndLN) of *Rnf5*^*−/−*^ mice (Supplementary Figure 5J). This data suggest that a higher fraction of migratory TdLN DCs are present in tumor-bearing *Rnf5*^*−/−*^ mice. These data indicate that DCs in the gut of *Rnf5*^*−/−*^ mice are quantitatively and functionally induced, underlying the stronger activation of the immune response seen in these mice. In all, these data establish that RNF5 plays an important role in control of intestinal DC recruitment and, consistent with previous reports^27,28^, the recruitment of DCs underlies T-cell activation and antitumor immunity.

### Reduced UPR coincides with altered microbiota and immunity

**Fig. 6:**
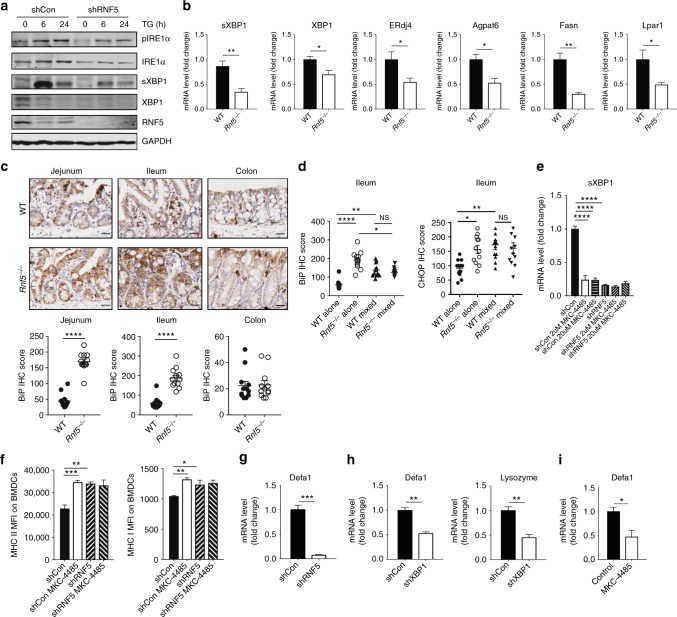
IEC of *Rnf5*^*−/−*^ mice activate immune responses and changes AMPs expression by IRE1α/sXBP1 signaling. **a** Western blot analysis of the indicated proteins in lysates of MODE-K-shCon and MODE-K-shRNF5 cells treated with thapsigargin (TG, 1 μM) for indicated time. **b** qRT-PCR analysis of mRNA levels of sXBP1, XBP1 and target genes in small intestine-derived IECs from naive WT or *Rnf5*^*−/−*^ mice (*n* = 4). **c** Representative micrographs of immunohistochemical (IHC) staining of BiP in the jejunum, ileum, and colon of YUMM1.5 tumor-bearing WT or *Rnf5*^*−/−*^ mice (scale bar = 25 μm). Lower graphs show quantification of IHC staining (*n* = 12 fields per group). Staining was scored semi-quantitatively based on staining intensity (0, 1, 2, or 3) multiplied by the percentage of positively stained cells (0–100), to give a maximum IHC score of 300. **d** Quantification of BiP and CHOP IHC staining in ileum sections from WT and *Rnf5*^−/−^ mice co-housed for 4 weeks prior to tumor inoculation (*n* = 12). **e** qRT-PCR analysis of sXBP1 mRNA levels in MODE-K-shCon and MODE-K-shRNF5 cells treated with the indicated concentration of MKC-4485 for 24 h (*n* = 3). **f** MFI of MHC class I and II on bone marrow-derived dendritic cells (BMDCs) incubated with media alone (no stimulation), conditioned media (CM) from shControl or shRNF5 MODE-K cells treated with MKC-4485 (*n* = 4). **g** qRT-PCR analysis of Defa1 mRNA levels in MODE-K-shCon and MODE-K-shRNF5 cells (*n* = 3). **h** qRT-PCR analysis of AMPs mRNA levels in IECs in MODE-K-shCon and MODE-K-shXBP1 cells (*n* = 3). **i**, qRT-PCR analysis of Defa1 mRNA levels in MODE-K cells treated with 2 μM MKC-4485 for 24 h (*n* = 3). Data are representative of three independent experiments (**b**, **e**–**i**) and two independent experiments (**a**, **c**, **d**) ≥3 mice per group. Graphs show the mean ± s.e.m. *P < 0.05, **P < 0.005, ***P < 0.001, ****P < 0.0001 by two-tailed *t* test or Mann–Whitney *U* test (**b**, **c**, **g**–**i**) or one-way ANOVA with Tukey’s correction for multiple comparisons (**d**–**f**)

Given that RNF5 functions in the ER stress and ERAD pathways^29,30^, we analyzed expression of ER-stress markers and related UPR pathways. CRISPR/Cas9-mediated RNF5 deletion in mouse melanoma cells markedly reduced ER-stress-mediated activation of inositol-requiring enzyme 1α (IRE1α) as seen by the level of sXBP1, key UPR components (Supplementary Figure 6A, B). To determine whether altered UPR signaling also occurs in IECs, we inhibited RNF5 expression (shRNF5) in MODE-K cells and then treated them with thapsigargin (TG). Reduced expression of X-box-binding protein 1 (XBP1) and sXBP1 as well as XBP1 target genes including endoplasmic reticulum-localized DnaJ4 (ERdj4)^31^ and triglyceride biosynthetic genes^32^, was seen in RNF5-depleted compared with expressing cells (Fig. 6a; Supplementary Figure 6C). Notably, IECs isolated from *Rnf5*^−/−^ mice also exhibited markedly reduced XBP1 and target gene mRNA levels (Fig. 6b). Similarly, a significant reduction in expression of key UPR components, including sXBP1, was observed following ER stress stimuli in *Rnf5*^−/−^ bone marrow-derived macrophages (BMMs) (Supplementary Figure 6D). Additionally, no obvious change was identified in the STING signaling pathway in a number of cell types, including BMMs, BMDCs (Supplementary Figure 6E, F). Of note, the reduction in UPR signaling components was accompanied by increased expression of ER-stress markers in the intestine, including the key chaperone-binding immunoglobulin protein (BiP) (Fig. 6c), suggesting that specific deregulation of UPR pathways (i.e., through IRE1α/sXBP1) can be partially compensated for by induced expression of other UPR pathway components (such as ATF6/BiP), consistent with previous reports^33,34^. Notably, the elevated BiP and CHOP expression seen in IECs was attenuated when *Rnf5*^−/−^ mice were co-housed with WT mice (Fig. 6d), further pointing to the effect of gut microbiota composition on UPR signaling.

To directly assess whether changes in UPR are able to trigger changes in immune response components, we treated MODE-K cells with IRE1α inhibitor (MKC-4485^35^). Administration of MKC-4485 caused inhibition of sXBP1 expression (Fig. 6e), and supernatant from these MODE-K cells was capable of activating DC (Fig. 6f), resembling those observed in MODE-K cells that were subjected to shRNF5. These results provide important support for the role of reduced IRE1α activity in the activation of DC and T cells, underlying the antitumor immunity seen in *Rnf5*^−/−^ mice.

Moreover, the expression pattern of several antimicrobial peptides, implicated in the regulation of gut microbiota composition, was markedly reduced in RNF5 or XBP1-depleted MODE-K cells (Fig. 6g, h), or MODE-K cells treated with MKC-4485 (Fig. 6i), similar to that observed in *Rnf5*^−/−^ mice. These data support the notion that altered UPR in *Rnf5*^−/−^ mice induce changes in both the gut microbiome and immune response.

### Reduced UPR in patients that respond to immunotherapy

**Fig. 7:**
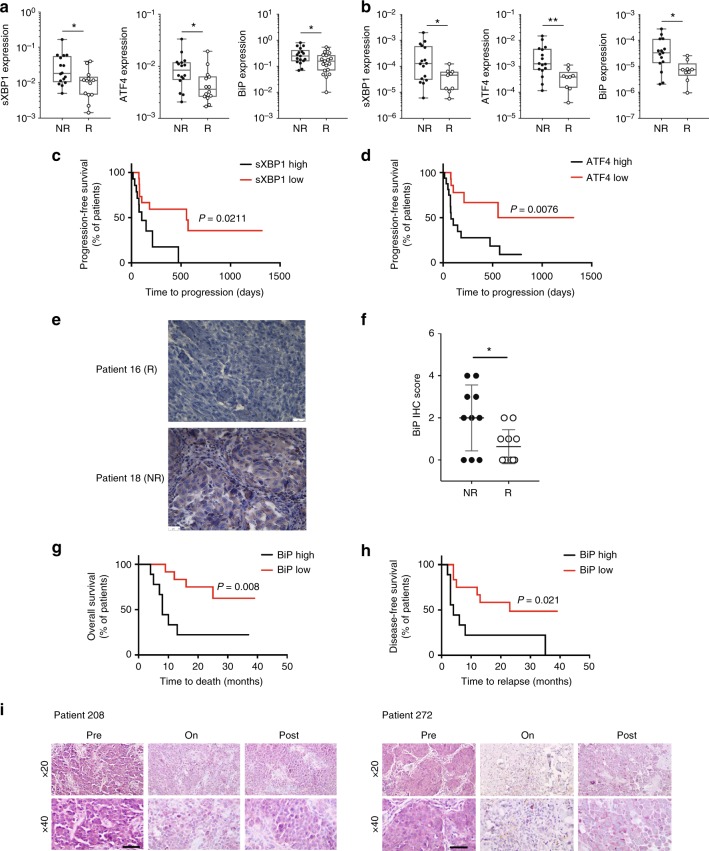
Altered UPR in melanoma responders of anti-CTLA-4 immune therapy. **a** Analysis of ER stress markers genes in non-responders (NR) (*n* = 16) and responders (R) (*n* = 14) to immune checkpoint therapy in melanoma samples from MGH/DFCC (USA). **b** Analysis of ER stress markers genes in NR (*n* = 16) and R (*n* = 9) to anti-CTLA-4 immunotherapy in melanoma samples from Zurich Hospital (Switzerland). **c** Kaplan–Meier curves for progression-free survival probability in melanoma patients demonstrating low (*n* = 15 patients) versus high sXBP1(*n* = 14 patients) in tumor specimens obtained before immune checkpoint therapy at MGH/DFCC. **d** Kaplan–Meier estimates of progression-free survival probability in melanoma patients demonstrating low (*n* = 14 patients) versus high ATF4 (*n* = 16 patients) in tumor specimens obtained before immune checkpoint therapy in melanoma samples at MGH/DFCC. **e** Representative micrographs of IHC staining of BiP after anti-PD-1 immunotherapy in melanoma samples obtained at Rambam Health Care Center (Israel) (scale bar = 25 μm). **f** IHC score of BiP staining in NR (*n* = 10) and R (*n* = 11) to anti-PD-1 immunotherapy in melanoma samples from Rambam Health Care Center (Israel). **g**, **h** Kaplan–Meier estimates of (**g**) overall survival and (**h**) disease-free survival probability in melanoma patients demonstrating low (<25%, *n* = 12 patients) versus high melanoma cell expression of BiP (>25%, *n* = 9 patients) in tumor biospecimens obtained before initiation of systemic anti-PD-1 antibody treatment in melanoma samples from Rambam Health Care Center (Israel). **i** IHC staining of BiP in melanoma samples from select patients prior to, on and after immunotherapy treatment from MGH/DFCC (USA). Scale bar = 50 μm. Graphs show the mean ± s.e.m. *P < 0.05, **P < 0.005, ***P < 0.001, ****P < 0.0001 by two-tailed *t-*test or Mann–Whitney *U* test (**a**, **b**, **f**) or log-rank test (**c**, **d**, **g**, **h**)

We next set to determine whether altered expression of UPR components can be also found in murine melanomas and patients that responded to immune checkpoint therapy. First, the expression of ER stress markers was assessed in murine melanomas collected following treatment with isotype or anti-CTLA4 antibodies. Notably, responders to anti-CTLA4 treatment exhibit reduced expression of sXBP1 and ATF4 (Figure S7A). Reduced expression of the chaperon BiP was also observed in responders to anti-CTLA4 treatment, compared with the isotype group (Supplementary Figure 7A). Furthermore, inhibition of XBP1 in mouse melanoma cells also coincided with a better response to anti-PD-1 treatment (Supplementary Figure 7B). Next, we analyzed the expression of ER stress marker genes in samples collected from melanoma patients in two independent cohorts (MGH and Zurich), prior to immune checkpoint therapy. Significantly, reduced expression of sXBP1, ATF4, and BiP mRNA levels was observed in responders to immune checkpoint therapy, compared with non-responders in both cohorts (Fig. 7a, b). Notably, further assessment of the MGH cohort revealed that patients with low sXBP1 expression exhibited a significant progression-free survival (median progression-free survival: 555 days vs. 103 days, *p* = 0.021, Fig. 7c), when compared with melanoma patients with high sXBP1 levels in the pre-treatment tumor specimens. Similarly, Kaplan–Meier survival curves revealed that low ATF4 expression levels was associated with a better progression-free survival (Fig. 7d). In a third independent cohort, biopsies of 21 patients who received Nivolumab/Pembrolizumab at Rambam Health Care Campus, with no prior or concurrent systemic therapy, were analyzed. In this cohort, low expression of BiP also correlated with good response to immunotherapy, whereas high BIP levels were found only in non-responders (*p* < 0.05). Notably, several biopsies taken at different time points from the same patient exhibited a consistent level of BiP expression (Fig. 7e, f). Patients with low BiP expression (< 25% of melanoma cells) prior to treatment exhibited a significantly better overall survival (median overall survival: high BiP group, 8 months; low BiP group, mostly alive, *p* = 0.008, Fig. 7g) and >5-fold increase in disease-free survival (median progression-free survival: 23 months vs. 4 months, *p* = 0.021, Fig. 7h) when compared with melanoma patients that had high BiP levels (>25% of melanoma cells). Furthermore, we analyzed BiP expression in paired biopsies obtained from patients prior to immune checkpoint therapy (pre-treatment), during treatment (on-treatment), and after the completion of immune checkpoint therapy (post-treatment). BiP expression was notably lower in 3 of 5 on- and post-treatment tumor biopsies, compared with the paired pre-treatment tumor biopsies (Fig. 7i). While in line with the finding in *Rnf5*^−/−^ mice, these findings suggest a relationship between ER stress markers and response to immunotherapy, thereby indicating the possible use of reduced sXBP1 and BiP expression as markers for melanoma patients responsiveness to immunotherapy.

## Discussion

Our data demonstrate that altered UPR signaling coincides with alteration of gut microbiota and activation of antitumor immunity. Cooperation and likely cross-talk between these two major components is expected to enable efficient tumor growth inhibition, exemplified in the *Rnf5*^−/−^ mice. Evidence for the activity of both gut microbiota and immune response components in limiting tumor growth comes from several observations. First, bone marrow transplant experiments revealed that antitumor immune cell phenotypes seen in *Rnf5*^−/−^ mice were evident only when bone marrow was transferred to *Rnf5*^−/−^ and not WT mice, confirming the role of a non-hematopoietic components that we subsequently identified as the altered microbiota composition. Notwithstanding, selective inactivation of RNF5 in specific cell types, (i.e., IEC), is expected to enable further mapping of mechanism underlying this phenomenon. Second, fecal transfer or administration of specific bacterial strains from *Rnf5*^−/−^ to WT GF mice recapitulated antitumor immunity phenotypes, supporting the impact of the microbiome on these outcomes. Third, *Rnf5*^−/−^ mice exhibit intrinsic inflammation, reflected by elevated serum cytokine levels, reduced intestinal villi length, increased crypt depth, and IECs cell death. Moreover, the expression pattern of a number of AMPs, implicated in the regulation of gut microbiota composition, was markedly reduced in *Rnf5*^−/−^ mice. These changes are expected to contribute to intestinal dysbiosis in *Rnf5*^−/−^ mice. Finally, deregulation of the IRE1/sXBP signaling pathway in IECs of *Rnf5*^−/−^ mice have altered DC and T-cell activation and AMPs expression.

We show that lack of RNF5, or inhibition of IRE1α or sXBP1, in intestinal epithelial cells is sufficient to increase CCL5 production, impacting DC recruitment and activation of T cells. Concomitant with the increased inflammasome and immune cell activation are changes in the expression of a repertoire of AMPs, implicated in defining the microbiota landscape, seen upon KD of RNF5 in MODE-K cells and in IEC isolated from *Rnf5*^−/−^ mice. Changes seen in IEC may underlie host-based mechanism, which defines the landscape of bacterial commensals and also trigger antitumor immunity. Notably, our findings suggest that changes in both microbiota and the immune system are required to achieve effective antitumor immunity to limit melanoma growth. Co-housing experiments and bacterial feeding to GF C57BL/6 mice and ASF-bearing C3H/HeN mice studies reveal the primary role of gut microbiota in defining the antitumor immunity phenotype. Enhanced DC number and activity were identified in the gut of *Rnf5*^−/−^ mice, reflected in (i) increased CD11c^+^ DCs seen in the  PP of tumor-bearing *Rnf5*^−/−^ mice, (ii) production of more cytokines and chemokines, (iii) greater induction of T cell cytokines by DCs from GALTs of tumor-bearing *Rnf5*^−/−^ mice, and (iv) enhanced frequency of CCR7^+^ DCs and mDCs in GALTs of tumor-bearing *Rnf5*^−/−^ mice, which coincides with greater migration properties. In a recent study^36^, we identified a significant increase in the frequency of total colonic DCs in colonic lamina propria cells from naive *Rnf5*^−/−^ mice compared with the WT genotype. Notwithstanding, our findings are in line with previous reports implicating altered UPR signaling in enhanced innate immunity associated with colitis and related inflammatory disorders^37,38^_._ IRE1α/β has also been implicated in regulating gut microbiota and determining colitis susceptibility^37^.

Our ability to identify a select subset of bacterial strains that are enriched in the *Rnf5*^−/−^ mice, which can elicit antitumor immunity and tumor growth inhibition when administered to GF WT mice, establish the importance of specific commensals in tumor growth control. The finding that commensal *B. rodentium* enhances antitumor immunity in vivo is consistent with effects reported for related family members (i.e., *B. fragilis, B. thetaiotaomicron*) ability to activate the immune system^4^.

Noteworthy, *Rnf5*^−/−^ mice exhibit basal intestinal inflammation, which develops into acute colitis following DSS administration^36^. S100A8, which was identified as a RNF5 substrate capable of mediating the acute colitis phenotype, and administration of neutralizing S100A8 antibodies blocked the colitis phenotypes^36^. While STING was previously reported to be a RNF5 substrate^9^, we did not observe RNF5-dependent changes in STING stability or STING-related signaling, consistent with the possibility that the regulation of STING may be mediated by other ubiquitin ligases^39–42^.

Reduced expression of sXBP1 and BIP was observed in human patient data—specifically in responders to immune checkpoint therapy, compared with non-responders, an observation that was made in different independent cohorts. Likewise, samples from a mouse melanoma model exhibit reduced UPR in those that responded to anti-CTLA4 therapy. Yet, while intestines of *Rnf5*^*−/−*^ mice also exhibited reduced sXBP1, they showed elevated BiP expression. These differences may well correspond to the tissue type examined, the time in which the analysis was performed (*Rnf5*^−/−^  mouse tumor samples were collected 2–3 weeks after tumor inoculation, whereas human tumor samples were obtained at different stages of their development, or following therapy) or exposure of human tumors microenvironmental factors not present in the *Rnf5*^−/−^ mice.

Overall, altered IEC immunogenicity and expression of antimicrobial peptides are expected to impact DC cell activation and microbiota composition, which together are expected to define the antitumor immunity and tumor growth inhibition, as seen in the *Rnf5*^−/−^ mice. Reduced UPR component expression was also seen in murine melanoma tumors that responded to CTLA4 therapy, and significantly, in patients that responded to immune checkpoint therapy, thus pointing the possible use of UPR components, sXBP1, ATF4, and BiP, as markers to stratify patients for these therapies. Our data also suggest that selected bacterial commensals associated with altered UPR signaling identified in the *Rnf5*^−/−^ mice could provide a novel approach to enhance antitumor activity. Consequently, defining the crosstalk between the UPR, gut microbiota, and immune checkpoint activity has significant potential expected to advance the development of novel therapeutic strategies.

## Methods

### Animals and tumor model

All experimental animal procedures were approved by the Institutional Animal Care and Use Committee of Sanford Burnham Prebys Medical Discovery Institute (approval # 13–130, 16–028, and 17–001) and complied with all relevant ethical regulations for animal testing and research. WT and *Rnf5*^−/−^ (originally generated in ref. ^43^) were generated from common *Rnf5*^+/−^ heterozygous, which were obtained from at least 10 generations backcross to WT C57BL/6 mice and were bred in house for several generations. All experiments used littermate controls, or their immediate descendants. *MyD88*^*−/*−^ mice were obtained with permission as gift from Dr. Shizuo Akira^44^. *Rnf5*^*−/*−^ and *MyD88*^−*/*−^ (C57BL/6) were crossed to generate *MyD88*^*+/*−^*Rnf5*^*+/*−^ heterozygous, and *MyD88*^−*/−*^*Rnf5*^*−/*−^ double-knockout mice were generated from *MyD88*^*+/−*^*Rnf5*^*+/−*^ heterozygous. In total, 6–14-week-old mice were used for all experiments^12^. Germ-free C57BL/6 mice and ASF-bearing C3H/HeN mice were bred and maintained at the University of Nebraska-Lincoln (UNL) Gnotobiotic Mouse Facility under gnotobiotic conditions in flexible film isolators. Experiments involving GF and gnotobiotic mice were approved by the Institutional Animal Care and Use Committee (IACUC) at UNL (protocol #1534). All mice were fed an autoclaved chow diet ad libitum (LabDiet 5K67, Purina Foods). Germ-free status was routinely checked as previously described^45^. Briefly, fresh feces were collected and analyzed by bacterial 16S rRNA gene-specific PCR (30 cycles, universal bacteria primers 8F and 1391R)^46^ in combination with aerobic and anaerobic culture of feces in Brain Heart Infusion, Wilkins-Chalgren and Yeast Mold broths, and on Tryptic Soy Agar plates (all media from Difco™ Becton Dickinson) at 37 °C for 7 days. ASF colonization status was verified by qPCR analysis of fecal samples as previously published^45^. Briefly, genomic DNA was extracted from fecal samples and ASF bacteria were quantified by qPCR with species-specific primers. Mouse selection for experiments was not formally randomized or blinded. For tumor growth experiments, mice were injected subcutaneously (s.c.) with 1 × 10^6^ tumor cells. Tumor size was measured twice a week for calculation of tumor volume. Tumors were weighed at the time of excision.

### Cell lines and gene silencing

Mouse melanoma cell lines used in this study include YUMM1.5, YUMM1.3, YUMM1.7, YUMM1.9 (Marcus Bosenberg, Yale University)^12^, B16F10 (ATCC), and B2905 (Glenn Merlino, NCI), and the mouse intestinal epithelial cell line MODE-K (Richard Blumberg Harvard Medical School), were cultured in the DMEM containing 10% FBS. All cell lines were free of mycoplasma and were authenticated. Cells were transfected with shRNAs using jetPRIME (PolyPlus). The RNAi Consortium lentiviral pLKO.1 control vector served as the shControl. The shRNAs were purchased from Sigma-Aldrich. Lentiviral particles were prepared using standard protocols. Briefly, shRNA plasmid and the second generation of packaging plasmids delta R8.2 and VSV-G (Addgene) were transfected into HEK293T cells. Viral supernatants were collected after 48 h of culture and used with polybrene (Sigma) for infection of indicated cell line.

### Quantitative real-time PCR amplification of 16S rRNA gene sequences

Fecal samples were weighed and bacterial DNA was extracted using the QIAmp Fast DNA Stool Mini Kit (Qiagen). The abundance of specific bacterial genes was amplified by qPCR with genera-specific 16S rRNA gene primers (Supplementary Table 6). The qPCR program started with an initial step at 95 °C for 5 min, followed by 35 cycles of 30S at 95 °C and 45S at 55 °C. The qPCRs were done using SYBR green supermix (Bio-Rad). Bacterial numbers were determined using standard curves with reference bacteria *E. coli* (ATCC). Standard curves were generated by qPCR using V3 primer pairs together with undiluted and a 10-fold dilution series of genomic template. The Ct values of each dilution were used to generate a standard curve of absolute abundance. qPCR measures the number of 16S rRNA gene copies per sample, not actual bacterial numbers.

### Bacterial strains and culture

The closest available bacterial strains matching those observed in *Rnf5*^*−/−*^ mice were identified. The 12 strains selected for administration as a cocktail in GF mice are shown in Supplementary Table 2. Lyophilized bacteria were resuspended and cultured anaerobically in the respective media formulations suggested by the vendors. Equal concentrations of each species, as measured by OD600, were pooled to make the bacterial cocktail. The Altered Schaedler Flora consisted of the following eight isolates: ASF 356, *Clostridium* sp.; ASF 360, *Lactobacillus intestinalis*; ASF 361, *Lactobacillus murinus*; ASF 457, *Mucispirillum schaedleri*; ASF 492, *Eubacterium plexicaudatum*; ASF 500, *Pseudoflavonifractor* sp.; ASF 502, *Clostridium* sp.; and ASF 519, *Parabacteroides goldsteinii*.

### Bacterial administration to germ-free mice

A cocktail of the bacterial species listed in Supplementary Table 2 or *B. rodentium* alone, was resuspended in PBS and administered via two oral gavages 1 day apart. Two weeks before tumor inoculation, each mouse was given 100 µl containing either a bacterial cocktail of 1.13 × 10^7^ organisms or 2.25 × 10^7^ *B. rodentium* alone along with 100 µl of cecal contents from ASF-bearing mice prepared as previously described. ASF cecal contents were harvested from ASF-bearing mice after euthanasia and homogenized in sterile 10% glycerol in phosphate-buffered saline (PBS) without calcium and magnesium, pH 7 (Corning Cellgro, Manassas, VA) at a ratio of 1-g cecal contents per 10 mL of 10% glycerol in PBS under rigorous vortexing. Inoculations were performed under sterile aerobic conditions in a biosafety cabinet.

### Bacterial DNA extraction and 16S library preparation

Mouse microbiota displays a stable homeostatic state when they are around 8 weeks^47^. In following this protocol, we did not collect microbiota earlier than 8 weeks. Mouse fecal pellets were frozen on dry ice, and stored at −80 °C. Bacterial DNA was extracted using the QIAmp Fast DNA Stool Mini Kit (Qiagen). To ensure efficient cell lysis, a 5-min bead-beating step using a Mini-Beadbeater-16 was included (Biospec Products, OK, USA). Library preparation for the Illumina MiSeq platform was performed by amplification of the V3–V4 region of the bacterial 16S ribosomal DNA gene using Forward primer: 5′-TCGTCGGCAGCGTCAGATGTGTATAAGAGACAGCCTACGGGNGGCWGCAG-3′ and reverse primer: 5′-GTCTCGTGGGCTCGGAGATGTGTATAAGAGACAG GACTACHVGGGTATCTAATCC-3′. Adapter and barcode sequences for dual indices were used as described by Illumina. PCR clean up steps were performed with QIAquick 96-PCR Clean up kit (Qiagen, Germany), and library quantification was performed using a KAPA Library Quantification Kit for Illumina platforms (KAPA Biosystems, MA, USA). An Experion Automated Gel Electrophoresis System (Bio-Rad, CA, USA) was used to measure the DNA concentration and purity of the pooled libraries. The 16S libraries were sequenced at the Analytical Genomics Core of Sanford Burham Prebys Medical Discovery Institute (Lake Nona, FL, USA) and Beijing Genomics Institute (Beijing, China).

### 16S sequencing data processing

The original FASTQ files from Illumina 250 basepair paired-end sequencing of the 120 samples (30 mice per group) were processed using a novel 16S amplicon sequencing pipeline HiMap (http://github.com/taolonglab/himap; *bioRxiv 565572*). The output of HiMap is Operational Strain Unit (OSU) which contains one or more bacterial strains that best match the 16S sequence and can not be further distinguished by it. The percentage similarity between the 16S sequence and the aligned region of 16S rRNA genes of the strains in the OSU is indicated. OSUs mapped to the same strains are grouped together (adding read counts), if the percentage similarities are within 2%. Read counts are converted into relative abundance as described in HiMap. Log10-transformed relative abundances were used for comparisons between samples under different experimental conditions.

### Taxa selection

Taxa that distinguished *Rnf5*^−/−^ mice microbiota from WT mice were selected as the overlap of the following three sets: (1) for each time point at day 0 (before tumor injection) and at day 24 (before tumor collection), Welch two-tailed *t* test was performed on the log10-transformed relative abundances of all OSU groups at the specified time point, and *p*-values were adjusted with Benjamini–Hochberg correction for multiple comparisons. OSU groups with adjusted *p*-value < 0.05 at both day 0 and day 24 were selected in this first set; (2) Wilcox rank-sum test was performed on relative abundances of all OSU groups at day 0 and day 24. OSU groups with adjusted *p*-value less than 0.05 at both day 0 and day 24 were selected in the second set; (3) the third set of OSU groups were selected by calculating spearman correlation between each of the OSU groups and tumor size at day 24 and keeping OSU groups with adjusted *p*-value <0.05. Initial OSU groups used in the above three tests are the ones with median relative abundance greater than 10^−4^ in at least one of the four groups: WT at day 0, *Rnf5*^−/−^ at day 0, WT at day 24, and *Rnf5*^−/−^ at day 24. The final set consists of 38 OSU groups that distinguished *Rnf5*^−/−^ mice microbiota from WT mice (Supplementary Table 1, Supplementary Figure 3C, D).

### In vivo antibody treatments

CD4^+^ or CD8^+^ T cells were depleted by intraperitoneal (i.p.) injection of 200 μg of anti-CD4 (clone GK1.5), anti-CD8 (clone 53–6.7), rat IgG2b isotype control, or rat IgG2a isotype control on days 0, 3, 6, 11, and 16 following tumor inoculation. The efficacy of depletion was confirmed by FACS analysis of blood samples. For anti-CTLA-4 or anti-PD-1 antibody treatment, mice were injected i.p. with 200 μg of anti-PD-1 (clone RMP1–14), anti-CTLA-4 (clone 9H10), Syrian hamster IgG isotype control (clone SHG-1), or rat IgG2a isotype control on days 7, 10, 13, and 16 after tumor inoculation. All mAbs for in vivo use were GoInVivo™ grade from BioLegend (San Diego, CA, USA).

### Mouse melanoma model subjected to anti-CTLA4 treatment

B2905 cells generated in Glenn Merlino Laborartory (NCI) were derived from a UV-induced melanoma in *Hgf*-tg mouse. Cells (1 × 10^6^) were subcutaneously implanted in the right flank of C57BL/6 mice. When tumors reached 75 mm^3^ in average, mice were randomized and anti-CTLA-4 (BioXCell, BE0164) or isotype control (BioXcell, BE0086) antibodies were administered i.v (final dose of 10 mg/kg). Treatment was done twice per week for four doses. Tumors were measured twice per week and collected 39 days post implantation when growth kinetics distinguished “responders” and “non-responders” to anti-CTLA-4.

### Bone marrow chimeras

WT or *Rnf5*^−/−^ recipient mice were lethally irradiated (1000 rads) and reconstituted by intravenous (i.v.) injection of 1 × 10^7^ bone marrow (BM) cells isolated from the femurs and tibias of donor WT or *Rnf5*^−/−^ mice. The recipient mice were treated with antibiotics (trimethoprim 8 mg/ml and sulfamethoxazole 40 mg/ml in the drinking water) for 3 weeks after injection. Reconstitution was confirmed 6–8 weeks after BM transfer, and the chimeric mice were then injected subcutaneously with 1 × 10^6^ YUMM1.5 melanoma cells.

### Tumor digestion

Tumors were excised, minced, and digested with 1 mg/ml collagenase D (Roche) and 100 µg/ml DNase I (Sigma) at 37 °C for 1 h. Digests were then passed through a 70-μm cell strainer to generate a single-cell suspension. The cells were washed twice with PBS containing 2 mM EDTA, and then stained for flow cytometry.

### Flow cytometry

Tumor-derived single-cell suspensions were washed twice with FACS staining buffer, fixed for 15 min with 1% formaldehyde in PBS, washed twice, and resuspended in FACS staining buffer.

For intracellular cytokine staining, cells were resuspended in complete RPMI-1640 (containing 10 mM HEPES, 1% nonessential amino acids and L-glutamine, 1 mM sodium pyruvate, 10% heat-inactivated fetal bovine serum (FBS), and antibiotics) supplemented with 50 U/mL IL-2 (NCI), 1 mg/mL brefeldin A (BFA, Sigma), and incubated with phorbol myristate acetate (10 ng/ml) and ionomycin (0.5 μg/ml) for 5 h at 37 °C. The cells were then fixed and permeabilized using a Cytofix/Cytoperm Kit (BD Biosciences) before staining.

Antibodies to the following proteins were obtained: CD45.2 (104), CD8α (53–6.7), CD4 (GK1.5), CD44 (IM7), PD-1 (RMP1–30), LAG-3 (C9B7W), TIM-3 (RMT3–23), CD45.1 (A20), TNF-α (MP6-XT22), IFN-γ (XMG1.2), CD11c (N418), CD11b (M1/70), MHC class II (M5/114.15.2), CD86 (GL1), CD40 (1C10), CD80 (16–10A1), PDCA (129c1), and B220 (RA3–6B2) were from BioLegend; antibodies to IL-2 (JES6–5H4) and MHC class I (AF6–88.5.5.3) were from eBioscience. All data were collected on an LSRFortessa (BD Biosciences) and analyzed using FlowJo Software (Tree Star). Gating strategies for immune cells analysis and cell sorting were provided in Supplementary Figure 8A–E.

### Histology and immunohistochemistry

Immediately after killing, the entire gastrointestinal tract was removed from the mice, split open lengthwise, rinsed, and rolled up from the proximal to distal end to form a “Swiss roll.” The tissues were fixed in 4% formalin overnight at 4 °C, washed with PBS, and embedded in paraffin. The embedded samples were sliced into 5-μm-thick sections and stained with H&E. For quantitative analysis, the villi length and the crypt depth were scored for each section. The score distributions between experimental groups were compared using proportional odds logistic regression using R software.

For immunohistochemistry, the tissue sections were deparaffinized, rehydrated, washed in PBS, subjected to antigen retrieval using Dako target retrieval solution, and incubated with 3% hydrogen peroxide for 30 min to quench endogenous peroxidase activity. The sections were then incubated overnight at 4 °C with an antibody to BiP (C50B12, Cell Signaling Technology) diluted 1:100 and BiP (ab21685, abcam) dilute 1:200 in Dako antibody diluent. Slides were then washed three times with PBS/Tween-20, incubated with Dako Labeled Polymer-HRP for 1 h at room temperature, washed again with PBS/Tween-20, developed with DAB, Bajoran Purple chromogen kit (BioCare) and with simple stain AEC solution (Histofine) and counterstained with hematoxylin. For mouse tissues, BiP staining was scored using a four tier intensity scale from 0 to 3 (low to high intensity) multiplied by the percentage of positively stained cells to generate an H score (maximum score of 300). For human melanoma tissues, specimens were scored as IHC 0, 1, 2, 3, or 4, if negative, ≥1% but <25%, ≥25% but <50%, ≥50% but <75%, or ≥75% of cells were BiP positive, respectively. All stained tissues were blindly evaluated by pathologists.

### Antibiotic treatments

Mice were treated for 2 weeks before tumor inoculation with ampicillin (1 mg/ml), neomycin (1 mg/ml), vancomycin (0.5 mg/ml), and metronidazole (1 mg/ml) (all Sigma-Aldrich) added to sterile drinking water. Solutions and bottles were changed 2–3 times per week.

### Western blotting

Cells were washed once with PBS at room temperature and resuspended in RIPA buffer (PBS containing 1% NP-40, 1% sodium deoxycholate, 1% SDS, 1 mM EDTA, and phosphatase and protease inhibitors). Lysates were sonicated on ice with a Microtip sonicator, centrifuged, and the supernatants were removed and subjected to SDS-PAGE. Proteins were transferred to nitrocellulose membranes (Osmonics Inc., MN, USA). Membranes were blocked and incubated with the respective primary antibodies followed by Alexa Fluor-conjugated secondary antibodies. The blots were imaged with an Odyssey detection system (Amersham Bioscience, NJ, USA). All uncropped blots are shown in Supplementary Figure 9.

### Antibodies and chemicals

The anti-RNF5 antibody was described previously^48^. Antibodies to IREα (3294) and sXBP1 (12782) were from Cell Signaling Technology, and antibodies to pIREα (ab48187) and XBP1 (ab28715) were from Abcam. Brefeldin A (BFA) and thapsigargin (TG) were purchased from Sigma-Aldrich. IRE1 inhibitor (MKC-4485) was kindly provided by Dr. John Patterson.

### Bone marrow-derived macrophages

Mice were euthanized with CO_2_, the femurs were removed, and the BM cells were harvested and washed. For macrophage differentiation, BM cells were resuspended in the RPMI-1640 medium containing 10% FBS, penicillin/streptomycin, and 2 mM L-glutamine and placed in Petri dishes. The supernatant from L929 cells (a source of macrophage-colony stimulating factor, M-CSF) was added at 30% (vol/vol), and the cells were incubated for 7 days. Differentiated macrophages were then harvested and used in the experiments.

### MODE-K cells stimulation with *B. rodentium*

MODEK-K cells were harvested, washed, and stimulated for 4 h with the growth medium alone or with medium containing rehydrated *B. rodentium* at a final ratio of 1:10 MODE-K cells to bacterial cells.

### BMDC activation and in vitro CD8^**+**^ T cell stimulation assay

To prepare conditioned media, MODE-K-EV or MODE-K-shRNF5 cells were incubated in DMEM supplemented with 10% FBS for 24 h at 37 °C to allow cell attachment. The cells were then treated with medium or MKC-4485 for 48 h in advanced DMEM (Thermo Fisher) containing 2 mM L-glutamine without serum. The medium was then collected and centrifuged at 2000 × *g* for 10 min at 4 °C to remove cellular debris. The resulting conditioned medium was used for treatment of BMDCs.

BM cells were isolated from the tibiae and femurs of WT C57BL/6 mice and cultured in the DMEM medium containing 10% FBS, 1% penicillin/streptomycin, and recombinant mouse GM-CSF (20 ng/ml; BioLegend) for 8 days at 37 °C. BMDCs were incubated for 24 h with the MODE-K conditioned medium (20% v/v) prepared as described above. OT-1 or P14 CD8^+^ T cells were incubated with 5 μM CFSE for 10 min at 37 °C and washed. Cells were then mixed 1:1 with the BMDCs and incubated for 72 h pulsed with 2 μg/ml of OVA peptide (SINFEKL) (AnaSpec) or GP33 peptide (AnaSpec). T-cell proliferation was monitored by CFSE dilution using flow cytometry and the division index (a measure of the average number of divisions which includes the undivided cells) was determined using FlowJo software (Tree Star). For intracellular cytokine staining, the cells were fixed, permeabilized with Cytofix/ Cytoperm (BD Biosciences), and stained with anti-TNF-α and anti-IFN-γ.

### Patient samples

Patients with melanoma provided written informed consent for the collection of tissue and blood samples for research and genomic profiling, as approved by the Dana-Farber/Harvard Cancer Center Institutional Review Board (DF/HCC Protocol 11–181), and the Kantonal Ethics Committee of Zurich (EK.647/800 & ZH.Nr.2014–0425) and Rambam Health Care Campus Institutional Review Board (RMB-0634–16131539). Fresh primary tumor specimens were obtained from patients prior to immunotherapy administration (see Supplementary Tables 3–5). Formalin-fixed tissue was analyzed to confirm that viable tumor was present via hematoxylin and eosin (H&E) staining and was used for immunohistochemical analysis and purification of RNA. Additional fresh tissue was processed immediately for purification of RNA. Of the MGH/Dana-Farber Cancer Center cohort, 36 of the 40 patients received immunotherapy for metastatic stage IV melanoma, 4 patients were given adjuvant therapy after definitive surgical resection. All patients classified as responders (R) showed clear radiographic decrease in disease and maintained an ongoing response without progression through to last follow-up. Patients classified as nonresponders (NR) did not respond to treatment radiographically and/or had clear and rapid progression. In the case of the five patients who received adjuvant therapy, R was defined as lack of post-treatment recurrence through to last follow-up. From the Zurich hospital cohort of 25 patients, all received immunotherapy for metastatic stage IV melanoma. All patients classified as R showed a reduction of tumor lesions within the first 12 weeks of treatment; whereas, NR had no measureable response to treatment at the first clinical end-point (12 weeks), as in Krieg et al.^49^. From the Rambam Health Care Center a cohort of 21 patients, all with metastatic melanoma received anti-PD-1 immunotherapy as a single agent, first-line systemic therapy was included in the study. In eight patients, more than one biopsy was examined. Response was assessed by a board certified oncologist (G.BS) who was blinded to the immunohistochemical results. All patients classified as responders (R) showed radiographic decrease of disease and maintained an ongoing response without progression through to last follow-up. Patients classified as nonresponders (NR) did not respond to treatment radiographically and/or had clear and rapid progression. For survival evaluation, the medical files of the patients were reviewed and tumor assessmemts were conducted according to Response Evaluation Criteria in Solid Tumors (RECIST), version 1.1, before treatment and then every 11–14 weeks until disease progression or death.

### RNA extraction and qRT-PCR analyses

The total RNA was extracted from mouse tumor samples individually using the RNeasy Fibrous Tissue Midi kit (QIAGEN), cells using GenElute (Sigma-Aldrich) or formalin-fixed paraffin-embedded (FFPE) patient samples using GenElute^TM^ (Sigma-Aldrich) FFPE RNA purification kit. Fresh patient tumor samples were homogenized and disrupted using a mortar and pestle followed by use of a QIAshredder. A QIAcube was used to harvest RNA from patient biopsies using the RNeasy Mini Protocol (Qiagen). The total RNA was reverse transcribed using high Capacity Reverse Transcriptase kits (Invitrogen) or the Superscript VILO cDNA Synthesis Kit (Invitrogen) according to the protocol by the manufacturer. Purity and concentration of extracted RNAs were checked and quantified by reading at 260 and 280 nm in a NanoDrop spectrophotometer (Thermo Fisher).

The qRT-PCR analyses were performed using Syber Green RT-PCR kits (Invitrogen) on a Bio-Rad CFX Connect Real-Time system or Roche LightCycler. Expression levels normalized to 18S or Tubb5 controls. Sequence-specific primers used in this study are shown in Supplementary Table 6.

### RNF5 gene deletion by CRISPR/Cas9 technology

RNF5-deficient cells were created using the CRISPR/Cas9 system^50^. A transient strategy was used to avoid nonspecific effects due to stable Cas9/sgRNA genome integration. YUMM1.7 cells were transiently transfected with a Cas9 and single-guide RNA (sgRNA) expression plasmid encoding GFP (Addgene plasmid #44719). The guide sequence was designed using the Optimized CRISPR Design at http://crispr.mit.edu: 5′-CGCTCGCGATTTGGCCCTTC-3′. After transfection, GFP-positive cells were sorted by FACS, cloned, and screened for *Rnf5* deletion by immunoblot analysis. Independent knockout clones and the control parental cells were analyzed as indicated.

### Isolation of intestinal epithelial cells

A 10 -cm section of mouse small intestine was opened longitudinally, minced, washed in 150 mM NaCl containing 1 mM DTT, and then resuspended in dissociation buffer (130 mM NaCl, 10 mM EDTA, 10 mM Hepes [pH 7.4], 10% FCS, and 1 mM DTT). The sections were incubated at 37 °C for 30 min with vigorous shaking to release the epithelial cells from the lamina propria. The epithelial cell suspension was then carefully aspirated, centrifuged, and washed in ice-cold PBS.

### Serum cytokine and chemokine detection

Cytokines and chemokines in the sera of tumor-bearing WT and *Rnf5*^−/−^ mice were quantified using the LEGENDplex^TM^ mouse inflammation panel and mouse proinflammatory chemokine panel (BioLegend), respectively. All data were collected on an LSRFortessa (BD Biosciences) and analyzed using LEGENDplex^TM^ software (BioLegend).

### NanoString nCounter assay

For each NanoString assay, an aliquot of 100 ng RNA was mixed with a NanoString code set mix and incubated at 65 °C overnight (16 h). The reaction mixes were loaded onto the NanoString nCounter Prep Station for binding and washing, and the resulting cartridge was transferred to the NanoString nCounter digital analyzer for scanning and data collection. Quantified expression data were analyzed using NanoString nSolver Analysis Software v2.0. After performing image quality control using a predefined cutoff value, we excluded the outlier samples using a normalized factor based on the sum of positive control counts greater than threefold. Data were normalized by scaling with the geometric mean of the built-in control gene probes for each sample.

### Bioinformatics analysis of the NanoString nCounter assay

For gene expression data from the NanoString nCounter assay, filtering of samples using quality control criteria was performed according to the manufacturer’s recommendations. Raw counts of samples passing quality control were normalized using 20 reference genes as internal controls (Abcf1, Alas1, Edc3, Eef1g, Eif2b4, G6pdx, Gusb, Hdac3, Hprt, Nubp1, Oaz1, Polr1b, Polr2a, Ppia, Rpl19, Sap130, Sdha, Sf3a3, Tbp, and Tubb5). Data were log2-transformed and used for further analysis. Student’s *t* test was applied to compare normalized expression values between groups. Ingenuity pathway analysis was used to interpret data in the context of biological processes, pathways, and networks.

### In vivo OT-1 T-cell proliferation assay

CD8^+^ T cells were isolated from the spleens of naive OT-1 CD45.1^+^ mice, labeled with CFSE, and injected i.v. into CD45.2^+^ WT or *Rnf5*^*−/−*^ mice (C57BL/6 background). After 24 h, the mice were injected s.c. with 1 × 10^6^ B16-OVA melanoma cells and the mice were left for 7 days. The spleen, tumor-draining lymph nodes, and non-draining lymph nodes were harvested and analyzed by flow cytometry. The proliferation of OT-1 CD8^+^ T cells was assessed by analysis of CFSE dilution within the population of gated CD45.1^+^ CD8^+^ T cells.

### Small intestine organoid culture

For small intestine organoid culture, crypt number was counted after isolation from tumor-bearing mice. A total of 500 crypts were mixed with 50 µl of Matrigel and 500 μl of organoid culture medium (Advanced DMEM/F12 containing 10 mM HEPES, 1× Glutamax, 1× N2 supplement, 1× B27 supplement, 50 ng/ml EGF, 1000 ng/ml R-spondin1, and 100 ng/ml Noggin).

### Statistical analysis

Unless otherwise noted, all data are shown as the mean ± s.e.m. Before statistical analysis, data were subjected to the Kolmogorov–Smirnov test to determine distribution. Variance similarity was tested using an F test for two groups and Bartlett’s test for multiple groups. Two groups were compared using the two-tailed *t* test for parametric data or the Mann–Whitney U test for nonparametric data. Multiple groups were compared using one-way ANOVA with Tukey’s, Dunnett’s, or Bonferroni’s correction for parametric data or using the Kruskal–Wallis test with Dunn’s correction for non-parametric data. Tumor growth curves were analyzed using two-way ANOVA with Sidak’s, Tukey’s, or Bonferroni’s correction for multiple comparisons. Kaplan–Meier estimates and the log-rank test were used to analyze statistical differences in overall and progression-free survival between melanoma patients treated with immunotherapy, whose pre-treatment tumor biopsies showed low versus high melanoma cell expression of BiP.

### Reporting Summary

Further information on experimental design is available in the Nature Research Reporting Summary linked to this article.

## Supplementary information

Reporting Summary

Supplementary Information

## Data Availability

The microbiome sequence data have been deposited in the National Center for Biotechnology Information (NCBI) Sequence Read Archive (SRA) under BioProject PRJNA524870. The gene expression data generated by the NanoString analysis has been deposited in the GEO database under the accession number GSE127753.

## Code availability

The custom code used to analyze 16S amplicon sequencing is publicly available at http://github.com/taolonglab/himap.
