# Supplementary material for: Gut microbiota dependent anti-tumor immunity restricts melanoma growth in Rnf5−/− mice
Source: Nat Commun. 2019 Apr 2;10:1492. doi: 10.1038/s41467-019-09525-y (PMC6445090; doi:10.1038/s41467-019-09525-y)
Supplement: Supplementary file 1 — Reporting Summary [file 41467_2019_9525_MOESM1_ESM.pdf]

nature research

Corresponding author(s): Ze'ev Ronai

Reporting Summary

Nature Research wishes to improve the reproducibility of the work that we publish. This form provides structure for consistency and transparency in reporting. For further information on Nature Research policies, see Authors & Referees and the Editorial Policy Checklist.

Statistical parameters

When statistical analyses are reported, confirm that the following items are present in the relevant location (e.g. figure legend, table legend, main text, or Methods section).

n/a	Confirmed	
☐	☒	The exact sample size (*n*) for each experimental group/condition, given as a discrete number and unit of measurement
☐	☒	An indication of whether measurements were taken from distinct samples or whether the same sample was measured repeatedly
☐	☒	The statistical test(s) used AND whether they are one- or two-sided<br>*Only common tests should be described solely by name; describe more complex techniques in the Methods section.*
☐	☒	A description of all covariates tested
☐	☒	A description of any assumptions or corrections, such as tests of normality and adjustment for multiple comparisons
☐	☒	A full description of the statistics including central tendency (e.g. means) or other basic estimates (e.g. regression coefficient) AND variation (e.g. standard deviation) or associated estimates of uncertainty (e.g. confidence intervals)
☐	☒	For null hypothesis testing, the test statistic (e.g. *F*, *t*, *r*) with confidence intervals, effect sizes, degrees of freedom and *P* value noted<br>*Give P values as exact values whenever suitable.*
☒	☐	For Bayesian analysis, information on the choice of priors and Markov chain Monte Carlo settings
☒	☐	For hierarchical and complex designs, identification of the appropriate level for tests and full reporting of outcomes
☒	☐	Estimates of effect sizes (e.g. Cohen's *d*, Pearson's *r*), indicating how they were calculated
☐	☒	Clearly defined error bars<br>*State explicitly what error bars represent (e.g. SD, SE, CI)*

Our web collection on statistics for biologists may be useful.

Software and code

Policy information about availability of computer code

Data collection	The microbiome sequence data have been deposited in the National Center for Biotechnology Information (NCBI) Sequence Read Archive (SRA) under BioProject PRJNA524870. The gene expression data generated by the NanoString analysis has been deposited in public access database, GEE127753. All other data supporting the findings of this study are available from the corresponding author upon reasonable request.
Data analysis	Data were analyzed using GraphPad Prism 6 and 7 (graphing, statistical analysis), FlowJo v10.1 and v9.9 (flow cytometry), nSolver Analysis Software (Nanostring data analysis), Aperio Imagescope (image acquisition).Customized algorithms were developed to detect and analyze bacterial taxa from 16S rRNA sequencing data. The algorithm HiMAP is specifically designed to analyze microbial 16S rRNA amplicon sequencing data with high accuracy and resolution. The source code of the algorithm will be available on GitHub before publication.

For manuscripts utilizing custom algorithms or software that are central to the research but not yet described in published literature, software must be made available to editors/reviewers upon request. We strongly encourage code deposition in a community repository (e.g. GitHub). See the Nature Research guidelines for submitting code & software for further information.

Data

Policy information about availability of data

All manuscripts must include a data availability statement. This statement should provide the following information, where applicable:

- Accession codes, unique identifiers, or web links for publicly available datasets
- A list of figures that have associated raw data
- A description of any restrictions on data availability

All the data are available within the article, supplementary information and supplementary data file or from the authors on request. The microbiome sequence data have been deposited in the National Center for Biotechnology Information (NCBI) Sequence Read Archive (SRA) under BioProject PRJNA524870. The gene expression data generated by the NanoString analysis has been deposited in public access database, GEE127753.

Field-specific reporting

Please select the best fit for your research. If you are not sure, read the appropriate sections before making your selection.

☒ Life sciences ☐ Behavioural & social sciences ☐ Ecological, evolutionary & environmental sciences

For a reference copy of the document with all sections, see nature.com/authors/policies/ReportingSummary-flat.pdf

Life sciences study design

All studies must disclose on these points even when the disclosure is negative.

Sample size	No statistical methods were used to predetermine sample size. For in vivo work, the number of animal used in each experiment was carefully estimated based on our preliminary experiments and previous experience. For in vitro experiments, a minimum of 3 samples were chosen as a sample size to ensure adequate power, unless stated otherwise.
Data exclusions	Samples that had undergone technical failure during processing were excluded from analyses.
Replication	Experiments were repeated at least 2 or 3 times independently (unless otherwise stated) and were reproducible.
Randomization	Samples were not randomized. Mice were chosen based on their genotype.
Blinding	The investigators were not blinded to group allocation during experiments and outcome assessment.

Reporting for specific materials, systems and methods

Materials & experimental systems

n/a	Involved in the study	
☐	☒	Unique biological materials
☐	☒	Antibodies
☐	☒	Eukaryotic cell lines
☒	☐	Palaeontology
☐	☒	Animals and other organisms
☐	☒	Human research participants

Methods

n/a	Involved in the study	
☒	☐	ChIP-seq
☐	☒	Flow cytometry
☒	☐	MRI-based neuroimaging

Unique biological materials

Policy information about availability of materials

Obtaining unique materials	All materials used are available from the authors or from standard commercial sources.

Antibodies

Antibodies used	BioLegend: anti-CD4 (clone GK1.5), anti-CD8 (clone 53-6.7), anti-PD-1 (clone RMP1-14), anti-CTLA-4 (clone 9H10), Syrian hamster IgG isotype control (clone SHG-1),antibodies to CD45.2 (104), CD8α (53-6.7), CD4 (GK1.5), CD44 (IM7), PD-1 (RMP1-30), LAG-3 (C9B7W), TIM3 (RMT3-23), CD45.1 (A20), TNF-α (MP6-XT22), IFN-γ (XMG1.2), CD11c (N418), CD11b (M1/70), MHC class II

(M5/114.15.2), CD86 (GL1), CD40 (1C10), CD80 (16-10A1), PDCA (129c1), and B220 (RA3-6B2.
eBioscience :IL-2 (JES6-5H4) and MHC class I (AF6-88.5.5.3)
Cell Signaling Technology: BiP (C50B12),IREα (3294) and sXBP1 (12782)
Abcam: BiP (ab21685), pIREα (ab48187), XBP1 (ab28715)
Antibodies are also listed in the Methods section under their respective experimental method

Validation

We chose these antibodies by the product data sheets, literature and our pilot studies.

Eukaryotic cell lines

Policy information about cell lines

Cell line source(s)

YUMM1.3, YUMM1.5, and YUMM1.9 cell lines were kindly provided by Marcus Bosenberg (Yale University). The B16-F10 mouse melanoma cell line was obtained from ATCC. MODE-K cell lines was established and kindly provided by Dr. Dominique Kaiserlian (Université Claude Bernard Lyon). B16-OVA cell line was provided by Dr. Richard W. Dutton (University of Massachusetts). Murine melanoma B2905 cells were derived in Glenn Merlino lab (NCI).

Authentication

Cell lines were obtained from verified sources, were not authenticated in our laboratory.

Mycoplasma contamination

All the cell lines tested negative for mycoplasma contamination (MycoAlert-Lonza)

Commonly misidentified lines
(See ICLAC register)

No commonly misidentified cell lines were used.

Animals and other organisms

Policy information about studies involving animals; ARRIVE guidelines recommended for reporting animal research

Laboratory animals

C57BL/6 WT and Rnf5-/- mice were maintained and used in Sanford Burnham Prebys Medical Discovery Institute. Germ-free C57BL/6 mice were bred and maintained at the University of Nebraska-Lincoln (UNL) Gnotobiotic Mouse Facility under gnotobiotic conditions in flexible film isolators. All mice used were a C57BL/6 genetic background and 6–12-weeks-old male mice. All experimental animal procedures were approved by the Institutional Animal Care and Use Committee of Sanford Burnham Prebys Medical Discovery Institute and UNL.

Wild animals

No wild animals were used in this study.

Field-collected samples

This study did not involve samples collected from the field.

Human research participants

Policy information about studies involving human research participants

Population characteristics

Patients with melanoma provided written informed consent for the collection of tissue and blood samples for research and genomic profiling, as approved by the Dana-Farber/Harvard Cancer Center Institutional Review Board (DF/HCC Protocol 11-181), and the Kantonal Ethics Committee of Zurich (EK.647/800 & ZH.Nr.2014-0425) and Rambam Health Care Campus Institutional Review Board (RMB-0634-16131539). Fresh tumor specimens were obtained from patients at baseline treatment with immunotherapy (see Table S8~Table S10). Formalin-fixed tissue was analyzed to confirm that viable tumor was present via hematoxylin and eosin (H&E) staining and was used for immunohistochemical analysis and purification of RNA. Additional fresh tissue was processed immediately for purification of RNA. Of the MGH/Dana-Farber Cancer Center cohort, 36 of the 40 patients received immunotherapy for metastatic stage IV melanoma, 4 patients were given adjuvant therapy after definitive surgical resection. All patients classified as responders (R) showed clear radiographic decrease in disease and maintained an ongoing response without progression through to last follow-up. Patients classified as non-responders (NR) did not respond to treatment radiographically and/or had clear and rapid progression. In the case of the 5 patients who received adjuvant therapy, R was defined as lack of post-treatment recurrence through to last follow-up. From the Zurich hospital cohort of 25 patients, all received immunotherapy for metastatic stage IV melanoma. All patients classified as R showed a reduction of tumor lesions within the first 12 weeks of treatment; whereas, NR had no measureable response to treatment at the first clinical end-point (12 weeks), as in Krieg et al16.   From the Rambam Health Care Center a cohort of 21 patients, all with metastatic melanoma received anti-PD-1 immunotherapy as a single agent, first line systemic therapy were included in the study. In 8 patients more than one biopsy was examined.   Response was assessed by a board certified oncologist (G.BS) who was blinded to the immunohistochemical results. All patients classified as responders (R) showed radiographic decrease of disease and maintained an ongoing response without progression through to last follow-up. Patients classified as non-responders (NR) did not respond to treatment radiographically and/or had clear and rapid progression. For survival evaluation the medical files of the patients were reviewed and tumor assessmemts were conducted according to Response Evaluation Criteria in Solid Tumors (RECIST), version 1.1, before treatment and then every 11-14 weeks until disease progression or death.

Recruitment

N/A

Flow Cytometry

Plots

Confirm that:

☒ The axis labels state the marker and fluorochrome used (e.g. CD4-FITC).

☒ The axis scales are clearly visible. Include numbers along axes only for bottom left plot of group (a 'group' is an analysis of identical markers).

☒ All plots are contour plots with outliers or pseudocolor plots.

☒ A numerical value for number of cells or percentage (with statistics) is provided.

Methodology

Sample preparation	Tumor-derived single-cell suspensions were washed twice with FACS staining buffer, fixed for 15 min with 1% formaldehyde in PBS, washed twice, and resuspended in FACS staining buffer. For intracellular cytokine staining, cells were resuspended in complete RPMI-1640 (containing 10 mM HEPES, 1% non-essential amino acids and L-glutamine, 1 mM sodium pyruvate, 10% heat-inactivated fetal bovine serum (FBS), and antibiotics) supplemented with 50 U/mL IL-2 (NCI), 1 mg/mL brefeldin A (BFA, Sigma), and incubated with phorbol myristate acetate (10 ng/ml) and ionomycin (0.5 ug/ml) for 16 h at 37°C. The cells were then fixed and permeabilized using a Cytofix/Cytoperm Kit (BD Biosciences) before staining.
Instrument	All data were collected on an LSRFortessa (BD Biosciences)
Software	All data were collected on FACSDive Software (BD) and analyzed using FlowJo v10.1 and v9.9 Software (Tree Star).
Cell population abundance	N/A
Gating strategy	CD4+ T cell analysis: CD45.2+CD4+CD44hiCD25-<br>CD8 T cell analysis: CD45.2+CD8+CD44hi<br>DCs analysis : CD45.2+CD11C+<br>pDC analysis: CD45.2+CD11Cint B220+PDCA+<br>mDC analysis: CD45.2+CD11C+B220-CD8α-CD11b+<br>CD8α+DC analysis: CD45.2+CD11C+B220-CD8α+CD11b-

☒ Tick this box to confirm that a figure exemplifying the gating strategy is provided in the Supplementary Information.
