# Supplementary material for: Gut microbiota dependent anti-tumor immunity restricts melanoma growth in Rnf5−/− mice
Source: Nat Commun. 2019 Apr 2;10:1492. doi: 10.1038/s41467-019-09525-y (PMC6445090; doi:10.1038/s41467-019-09525-y)
Supplement: Supplementary file 2 — Supplementary Information [file 41467_2019_9525_MOESM2_ESM.pdf]

Supplementary Information

Regulation of Gut Microbiota and Anti-Tumor Immunity in *RNF5*$^{-/-}$ mice Restricts Melanoma Growth

Li et al.

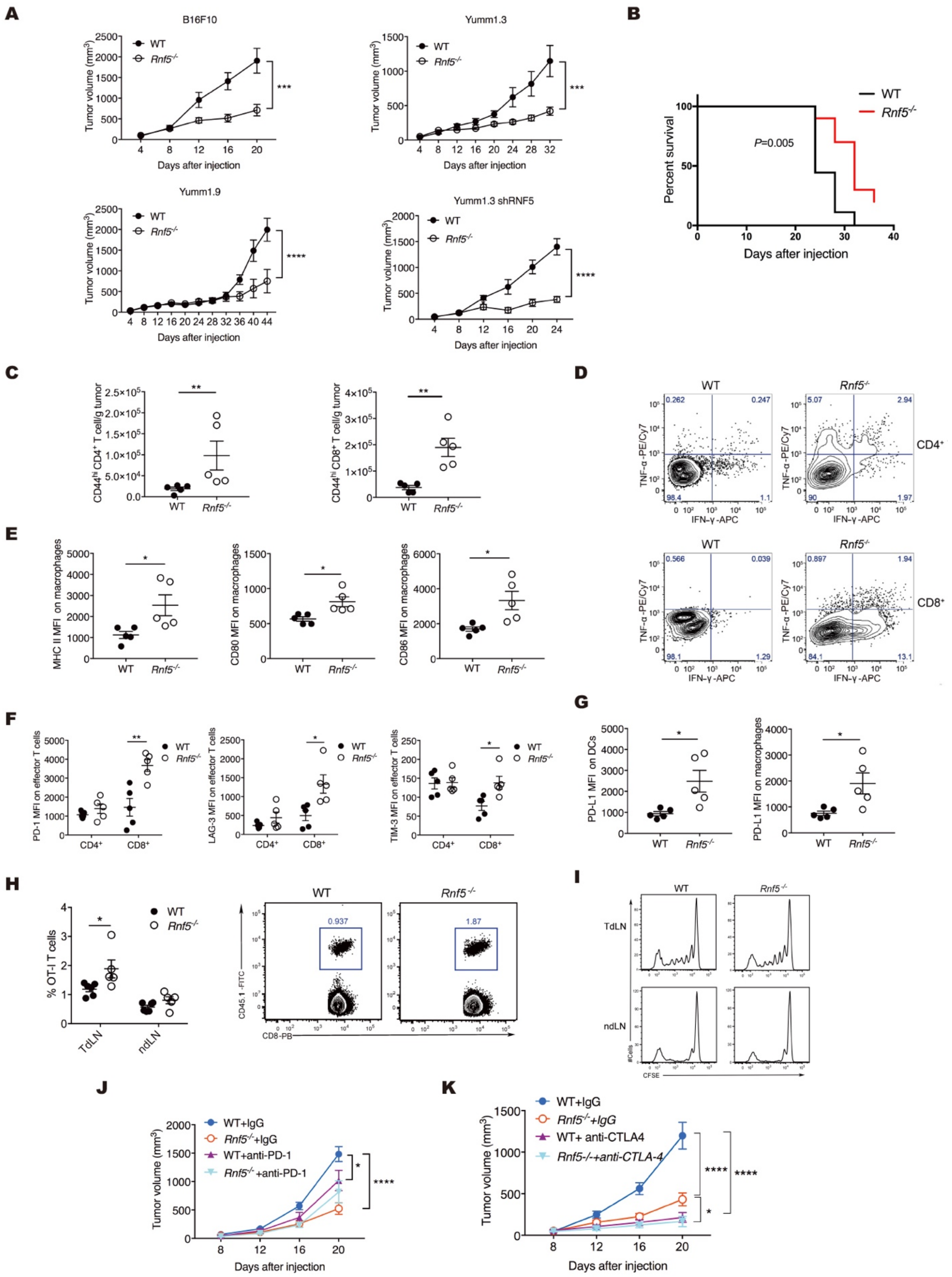
A
B16F10
Yumm1.3
Yumm1.9
Yumm1.3 shRNF5
WT
Rnf5-/-
Tumor volume (mm3)
Days after injection
***
****
B
Percent survival
P=0.005
Days after injection
C
CD44hi CD4+ T cell/g tumor
CD44hi CD8+ T cell/g tumor
**
D
TNF-α-PE/Cy7
IFN-γ-APC
CD4+
CD8+
0.262
0.247
98.4
1.1
5.07
2.94
90
1.97
0.566
0.039
98.1
1.29
0.897
1.94
84.1
13.1
E
MHC II MFI on macrophages
CD80 MFI on macrophages
CD86 MFI on macrophages
*
F
PD-1 MFI on effector T cells
LAG-3 MFI on effector T cells
TIM-3 MFI on effector T cells
CD4+
CD8+
G
PD-L1 MFI on DCs
PD-L1 MFI on macrophages
H
% OT-I T cells
TdLN
ndLN
CD45.1-FITC
CD8-PB
0.937
1.87
I
TdLN
ndLN
#Cells
CFSE
J
WT+IgG
Rnf5-/-+IgG
WT+anti-PD-1
Rnf5-/- +anti-PD-1
K
WT+IgG
Rnf5-/-+IgG
WT+ anti-CTLA4
Rnf5-/-+anti-CTLA-4

Supplementary Figure 1. Enhanced anti-tumor immune responses in *Rnf5*$^{-/-}$ mice. **A**, Growth of B16F10, YUMM1.9, YUMM1.3, and YUMM1.3-shRnf5 melanoma cells in WT and *Rnf5*$^{-/-}$ mice (n = 5). **B**, Survival of WT and *Rnf5*$^{-/-}$ mice bearing YUMM1.5 melanoma (WT, n = 9; *Rnf5*$^{-/-}$, n = 10). Loss of survival was defined as death or when tumor diameter reached or exceeded 2cm in any dimension or the tumor volume reached 2 mm^3. **C**, Quantification of tumor-infiltrating ($CD44^{hi}$) $CD4^+$ and $CD8^+$ T cells on day 16 after injection of 10^6 YUMM1.5 cells into WT and *Rnf5*$^{-/-}$ mice (n = 5). **D**, Representative flow cytometry plots of cytokine production by tumor-infiltrating $CD4^+$ and $CD8^+$ T cells. **E**, MFI of MHC class II, CD80, and CD86 in tumor-infiltrating macrophages on day 24 after injection of YUMM1.5 cells (n = 5). **F**, MFI of PD-1, LAG-3, and TIM-3 in tumor-infiltrating ($CD44^{hi}$) $CD4^+$ and $CD8^+$ T cells at day 24 after injection of YUMM1.5 cells (n = 5). **G**, MFI of PD-L1 in tumor-infiltrating DCs or macrophages on day 24 after injection of YUMM1.5 cells (n = 5). **H**, Frequency of $CD45.1^+CD8^+$OT-I T cells from TdLN or ndLN from WT or *Rnf5*$^{-/-}$ mice (WT, n = 6; *Rnf5*$^{-/-}$, n = 5). **I**, Representative FACS profiles showing dilution of CFSE staining in $CD45.1^+CD8^+$WT OT-1 T cells from TdLNs and ndLNs. **J**, Tumor growth after injection of YUMM1.5 cells into WT or *Rnf5*$^{-/-}$ mice treated with control IgG or anti-PD-1 antibody (IgG, n = 9; anti-PD-1, n = 8). **K**, Tumor growth after injection of YUMM1.5 cells into WT or *Rnf5*$^{-/-}$ mice treated with control IgG or anti-CTLA-4 blocking antibody (n = 9). Data are representative of three independent experiments (**A**, **D**-**G**), two independent experiments (**C**, **H**-**J**) and one experiment (**B**, **K**). Graphs show the mean ± s.e.m. *P < 0.05, **P < 0.005, ***P < 0.001, ****P < 0.0001 by two-way ANOVA with Sidak's or Bonferroni's correction (**A**, **J** and **K**) or or log-rank test (**B**). Individual mice were analyzed by two-tailed t-test or Mann–Whitney U test (**C**, **E**–**H**).

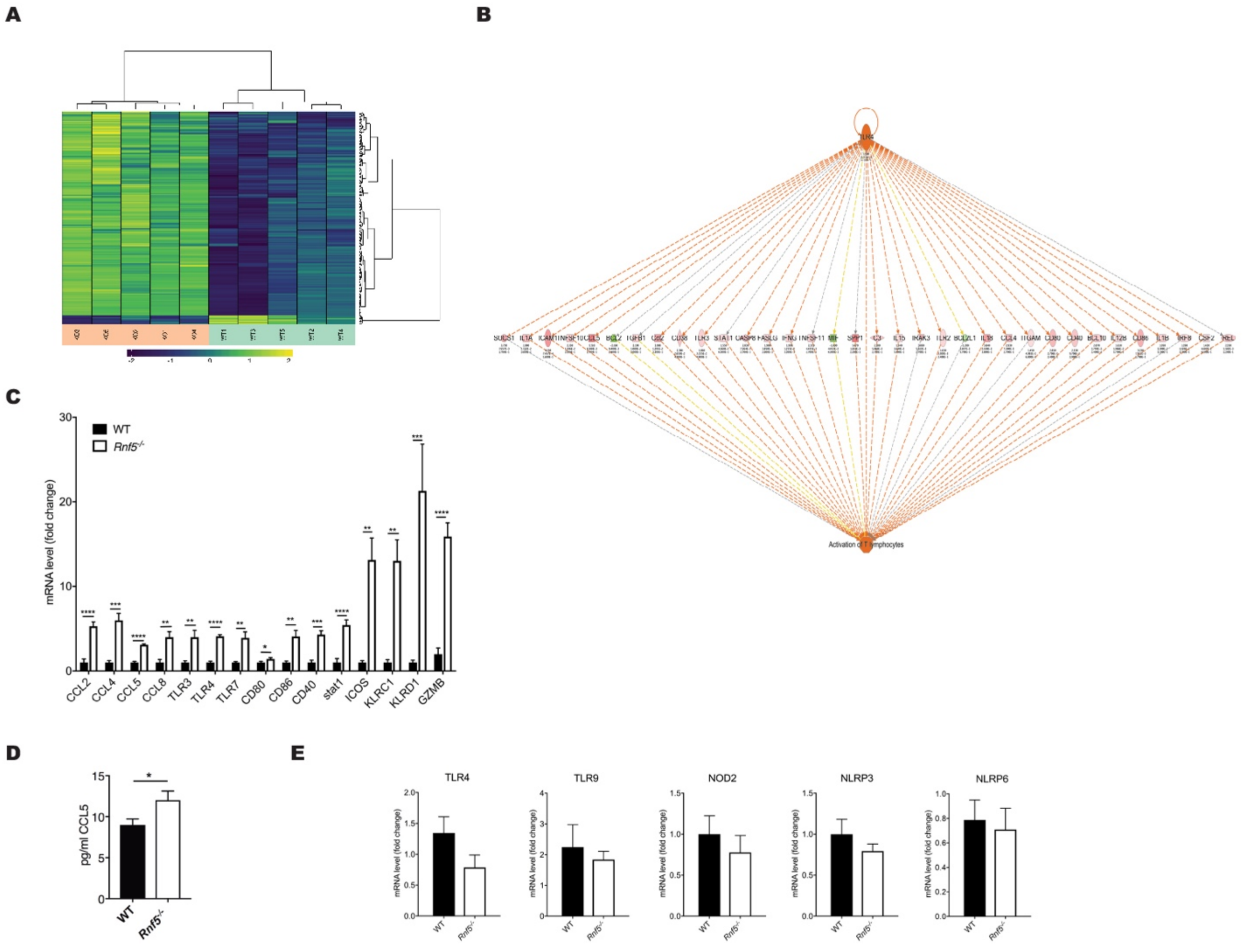

Supplementary Figure 2. Enhanced anti-tumor immunoregulatory gene expression in *Rnf5*$^{-/-}$ mice. **A,** NanoString analysis of PanCancer Immune Profiling genes in YUMM1.5 melanoma tumors from WT and *Rnf5*$^{-/-}$ mice. The heat map shows 403 genes with >1.2-fold difference ($P \leq 0.05$) in expression between the two strains (n = 5). **B,** Ingenuity pathway analysis of upstream regulators and effectors in tumors from *Rnf5*$^{-/-}$ mice compared with WT mice. **C,** qPCR validation of differentially expressed genes identified by gene expression profiling of tumors from WT and *Rnf5*$^{-/-}$ mice (n = 5). **D**, CCL5 levels in the serum of WT or *Rnf5*$^{-/-}$ mice 10 days after tumor inoculation (n = 5). **E**, qRT-PCR analysis of pathogen receptor mRNA levels in IECs from naïve WT or *Rnf5*$^{-/-}$ mice (n=4). Data are representative of three independent experiments (**C**) and two independent experiments (**D, E**). Graphs show the mean ± s.e.m. *$P < 0.05$, **$P < 0.005$, ***$P < 0.001$, ****$P < 0.0001$ by two-tailed t-test or Mann–Whitney U test.

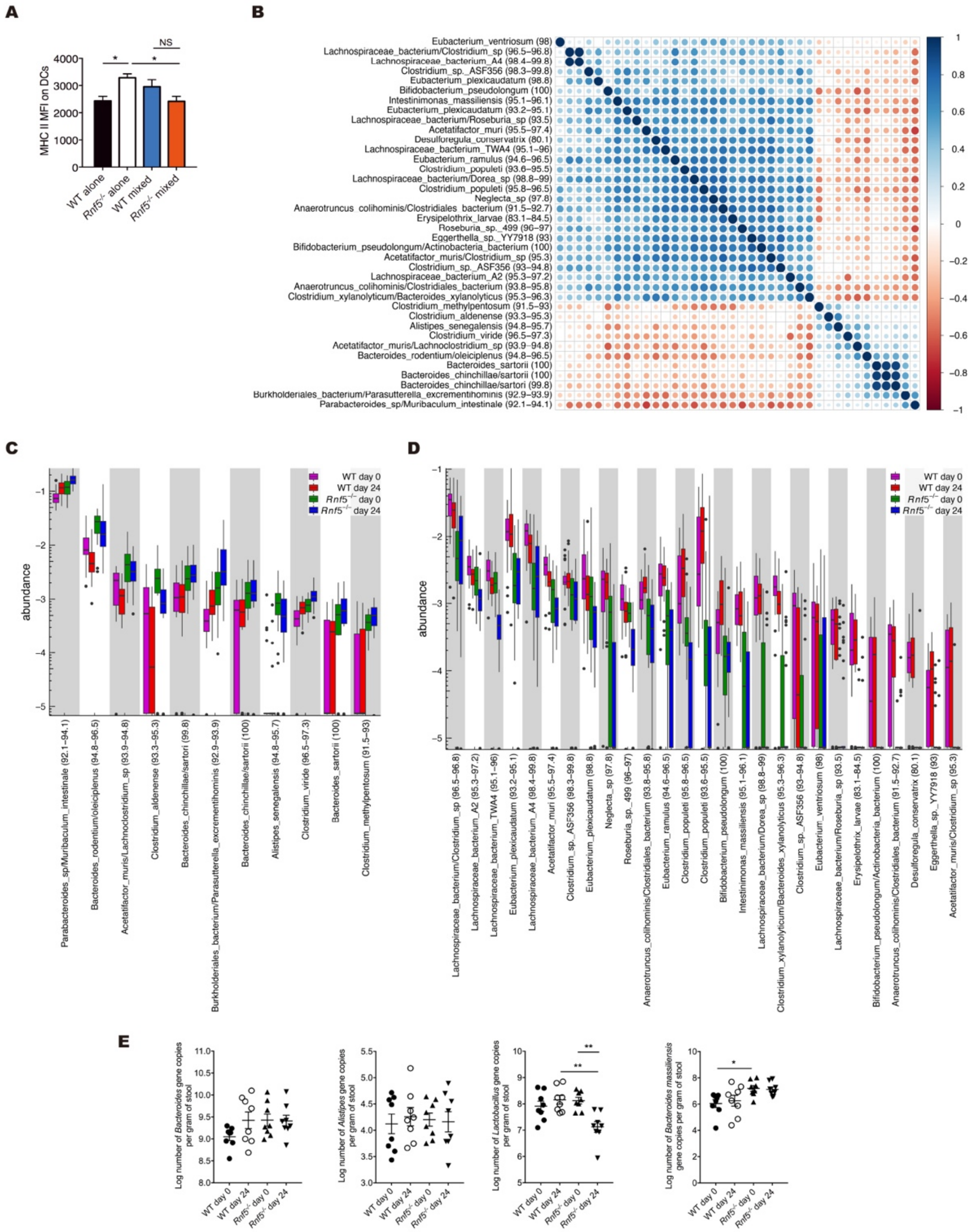

Supplementary Figure 3. Marked difference in microbial community structure between *Rnf5*–/– and WT mice. **A,** MFI of MHC class II on tumor-infiltrating DCs (CD45+ CD11c+) in tumor-infiltrating effector (CD44hi) CD8+ T cells from WT or *Rnf5*–/– mice housed alone or together (mixed) for 4 weeks prior to YUMM1.5 inoculation. Cells were analyzed on day 24 after inoculation (*n* = 10). **B**, Correlation heat map of the relative abundance of the 38 taxa. The color and size of the circles indicate Spearman's correlation for the relative abundance of taxa and the significance of the correlation, respectively (*n* = 60). **C,** Boxplot of the relative abundance of the 11 taxa enriched in *Rnf5*–/– mice microbiota that are negatively correlated with tumor size (*n* = 30). **D,** Boxplot of the relative abundance of the 27 taxa enriched in WT mice microbiota that are positively correlated with tumor size (*n* = 30). **E,** Fecal samples were weighed and DNA were extracted. Quantification of different bacterial copy number in WT and *Rnf5*-/- mice were performed on day 0 and day 24 (*n* = 8). Data are representative of two independent experiments (**A**, **E**). Graphs show the mean ± s.e.m. *P < 0.05, **P < 0.005, ***P < 0.001, ****P < 0.0001 by one-way ANOVA with Tukey's correction.

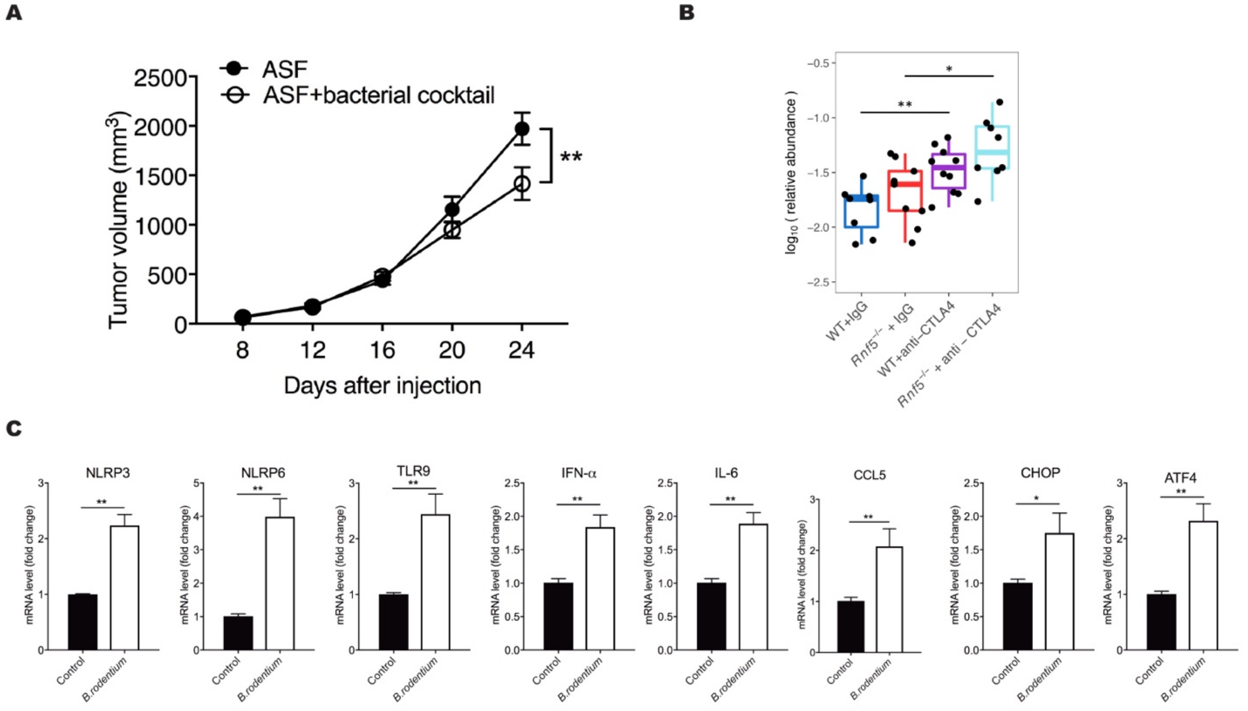

Supplementary Figure 4. *B. rodentium* induced ER stress signature and activated components of the TLR signaling pathway. **A**, SW1 tumor growth in ASF-bearing C3H/HeN mice undergoing oral gavage with or without bacterial cocktail prior to tumor inoculation (ASF, n = 15; ASF + bacterial cocktail, n = 14). **B**, Quantification of *B. rodentium* abundance on day 24 after tumor injection in WT or $Rnf5^{-/-}$ mice treated with control IgG or anti-CTLA-4 blocking antibody on days 7, 10, 13, and 16 after injection of YUMM1.5 tumor cells (n = 9). **C,** qRT-PCR analysis of the indicated ER stress-related and TLR signaling pathway mRNAs in MODE-K cells left untreated (control) or incubated with *B. rodentium* (n = 6). Data are representative of three independent experiments (**C**) and one experiment (**A**). Graphs show the mean ± s.e.m. *P < 0.05, **P < 0.005, ***P < 0.001, ****P < 0.0001 by two-tailed t-test or Mann–Whitney U test (**C**), one-way ANOVA with Tukey's correction for multiple comparisons (**B**) or two-way ANOVA (**A**).

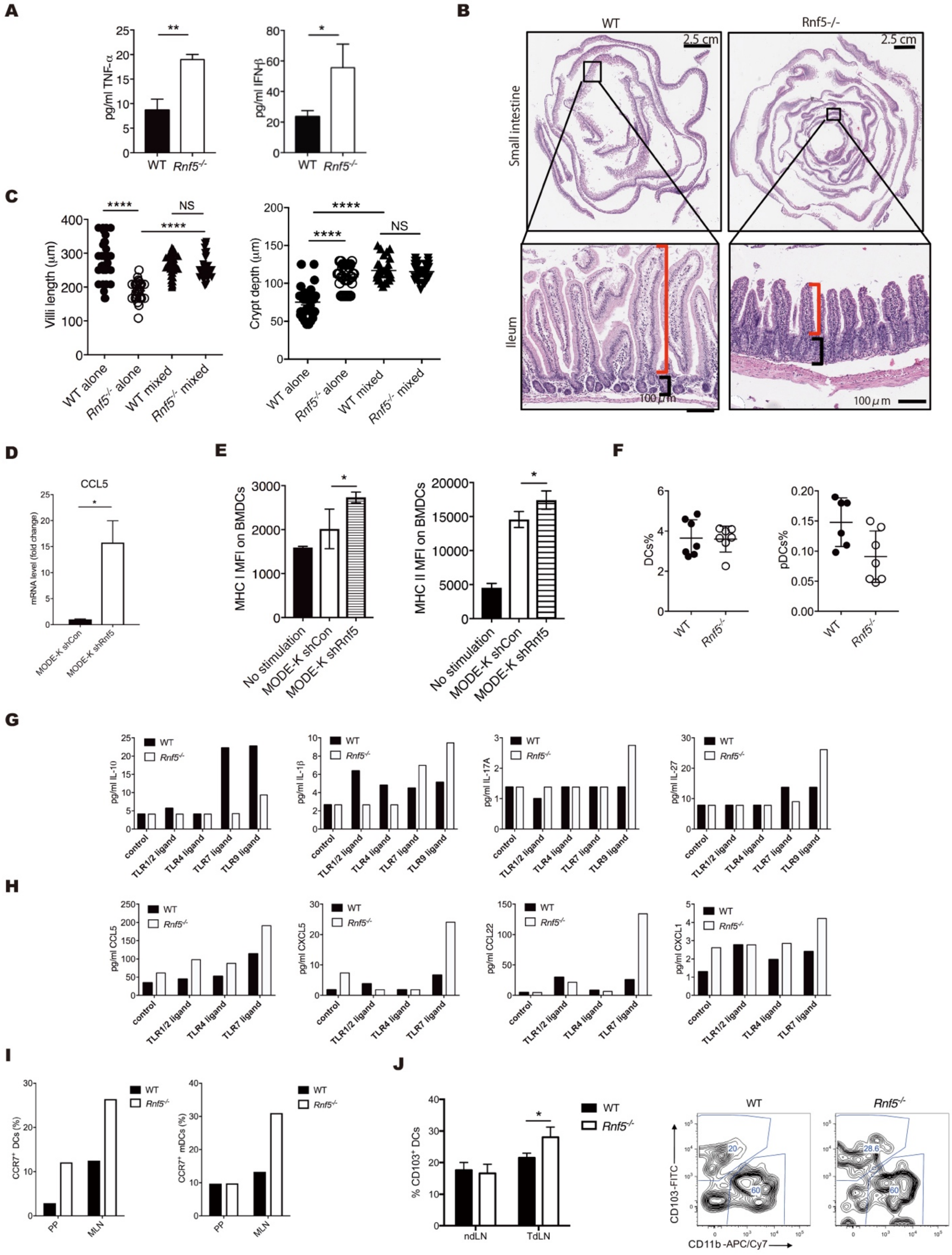
A
pg/ml TNF-α
WT
Rnf5-/-
**
pg/ml IFN-β
*
B
WT
Rnf5-/-
2.5 cm
Small intestine
Ileum
100 μm
C
Villi length (μm)
Crypt depth (μm)
****
NS
WT alone
Rnf5-/- alone
WT mixed
Rnf5-/- mixed
D
CCL5
mRNA level (fold change)
MODE-K shCon
MODE-K shRnf5
E
MHC I MFI on BMDCs
MHC II MFI on BMDCs
No stimulation
MODE-K shCon
MODE-K shRnf5
F
DCs%
pDCs%
G
pg/ml IL-10
pg/ml IL-1β
pg/ml IL-17A
pg/ml IL-27
control
TLR1/2 ligand
TLR4 ligand
TLR7 ligand
TLR9 ligand
H
pg/ml CCL5
pg/ml CXCL5
pg/ml CCL22
pg/ml CXCL1
I
CCR7+ DCs (%)
CCR7+ mDCs (%)
PP
MLN
J
% CD103+ DCs
ndLN
TdLN
CD103-FITC
CD11b -APC/Cy7
20
60
28.6

Supplementary Figure 5. Altered intrinsic inflammation in *Rnf5*$^{-/-}$ mice. **A**, Serum cytokines in naïve WT or *Rnf5*$^{-/-}$ mice (*n*=6). **B**, H&E-stained small intestine Swiss roll sections in YUMM1.5 tumor-bearing WT mice or *Rnf5*$^{-/-}$ mice (upper panels). Villi length (red bracket) and crypt depth (black bracket) were labeled in the lower panel (scale bar 100μm). **C**, Villi lengths and crypt depth in YUMM1.5 tumor-bearing WT and *Rnf5*$^{-/-}$ mice co-housed for 4 weeks prior to tumor inoculation (*n* = 32). **D**, qRT-PCR analysis of CCL5 mRNA levels in MODE-K-shCon and MODE-K-shRnf5 cells (*n* = 3). **E**, MFI of MHC class **I** and **II** on bone marrow-derived dendritic cells (BMDCs) incubated with media alone (no stimulation), conditioned media (CM) from shControl or shRNF5 MODE-K cells (*n* = 4). **F**, Percentage of total DCs and pDCs in Peyer's patches isolated from naïve WT or *Rnf5*$^{-/-}$ mice (*n* = 6). **G**, **H**, Average of cytokine and chemokine secretion by FACS-sorted $CD45^+$ $NK1.1^-$ $CD19^-$ $Thy1.2^-$ $CD11c^+$ DCs isolated from Peyer's patches from naïve (WT) and *Rnf5*$^{-/-}$ mice (pooled from *n* = 10/group). Cells were cultured with or without ligands for TLR1/2 (Pam3CSK4, 300 ng/ml), TLR4 (LPS, 1 μg/ml), TLR7 (R848, 10 μg/ml), and TLR9 (ODN1826, 5 μM) for 18 h, and the supernatants were removed for quantification of cytokines (**G**) and chemokines (**H**). **I**, 10 days after tumor injection, DCs from PP and MLN were isolated and pooled per group (*n*=10 mice/group). Frequency of $CCR7^+$ DCs and mDCs were detected. **J**, Frequency of $CD103^+$ DCs from TdLN and ndLN from WT and *Rnf5*$^{-/-}$ mice. Dot plots show gating $CD103^+CD11C^+MHC\ II^{high}$ cells (*n*=10). Data are representative of three independent experiments (**D**, **E**) and two independent experiments (**A**-**C**, **F-J**). Graphs show the mean ± s.e.m. *P < 0.05, **P < 0.005, ***P < 0.001, ****P < 0.0001 by two-tailed t-test or Mann–Whitney U test (**A**, **D, F, I and J**) or one-way ANOVA with Tukey's correction for multiple comparisons (**C** and **E**).

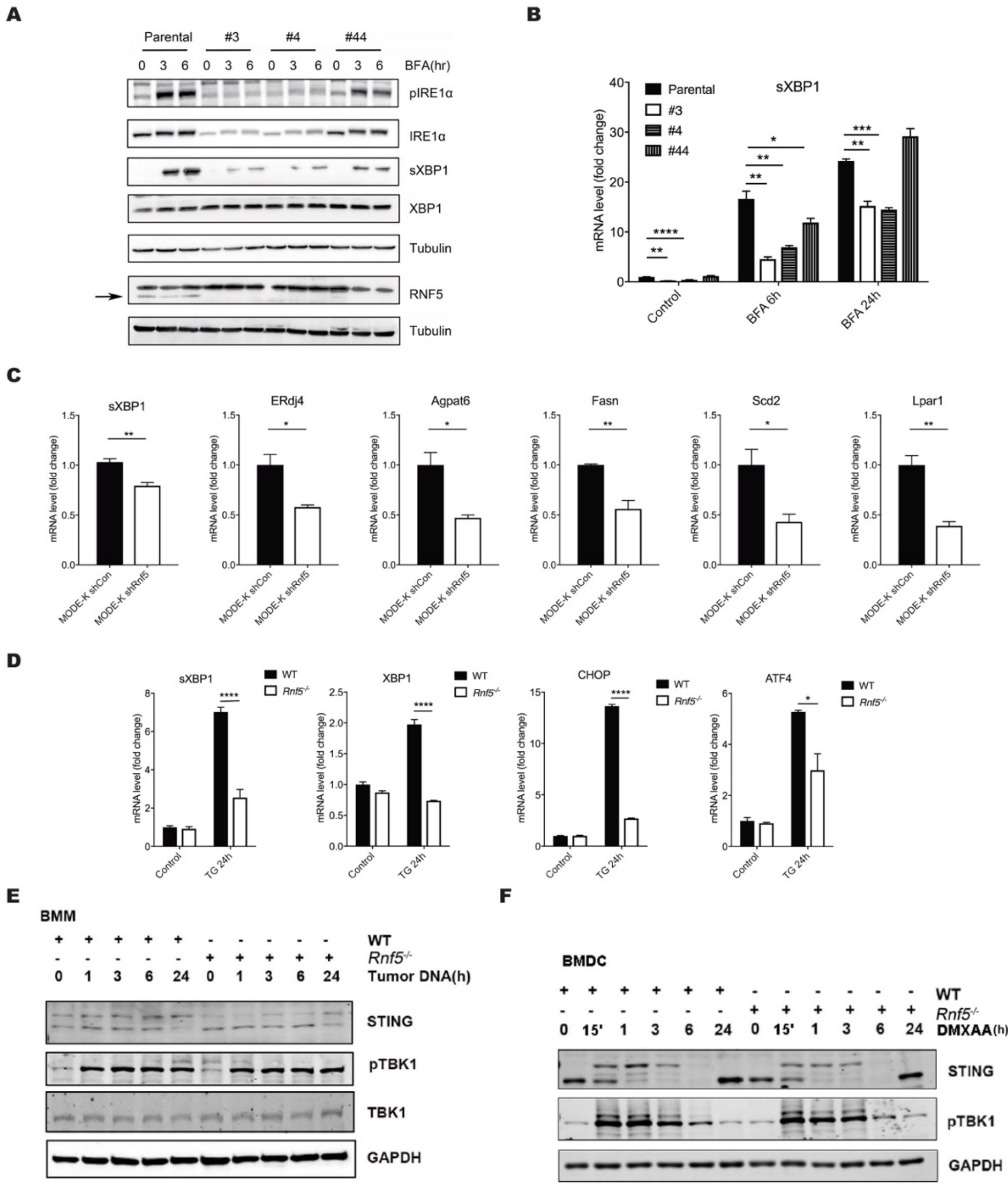

Supplementary Figure 6. Altered UPR in *Rnf5*$^{-/-}$ mice induce concerted changes in the gut microbiome and immune response. **A**, Western blot analysis of the indicated proteins in lysates of YUMM1.7 melanoma cells treated with 3μM BFA for indicated time. Parental, homozygote *Rnf5*$^{-/-}$ (two independent clones. #3 and #4), and a heterozygote *Rnf5*$^{+/-}$ clone (#44) are shown. **B**, qRT-PCR analysis of sXBP1 mRNA in the cells shown in (a) treated with 3μM BFA for indicated time. **C**, qRT-PCR analysis of mRNA levels of sXBP1, ERdj4, and indicated triglyceride-related genes in MODE-K cells expressing control or RNF5-specific shRNA. **D**, RT-PCR analysis of indicated ER stress-related mRNAs in bone marrow-derived macrophages (BMDMs) from WT and *Rnf5*$^{-/-}$ mice after treatment *in vitro* with or without 1μM thapsigargin (TG) for indicated times (n = 4). **E**, Western blot analysis of the indicated proteins in lysates of BMM treated with tumor DNA for indicated times (n = 3). **F**, As in panel E, except that BMDC were treated with DMXAA for indicated times (n = 3). Data are representative of three independent experiments (B, C, D) and two independent experiments (**A**, **E**, **F**). Graphs show the mean ± s.e.m. *P < 0.05, **P < 0.005, ***P < 0.001, ****P < 0.0001 by two-tailed t-test or Mann–Whitney U test (**C**-**D**) or one-way ANOVA with Tukey's correction for multiple comparisons (**B**).

A

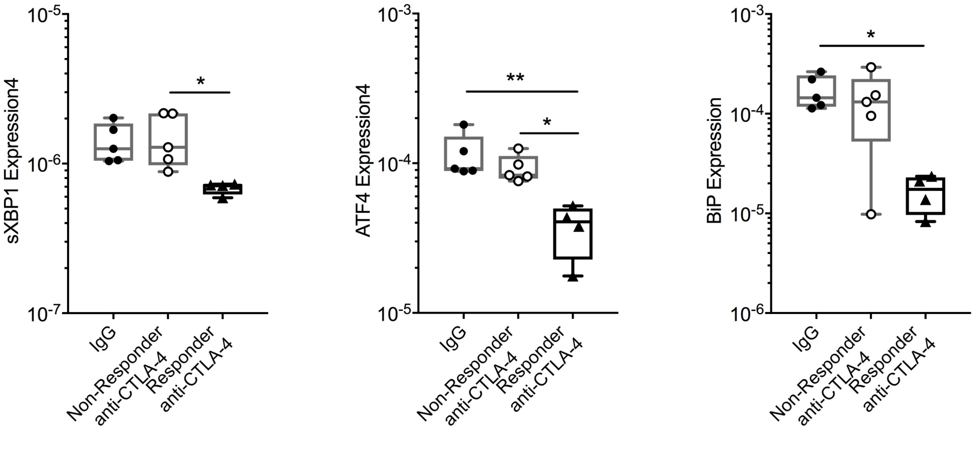

B

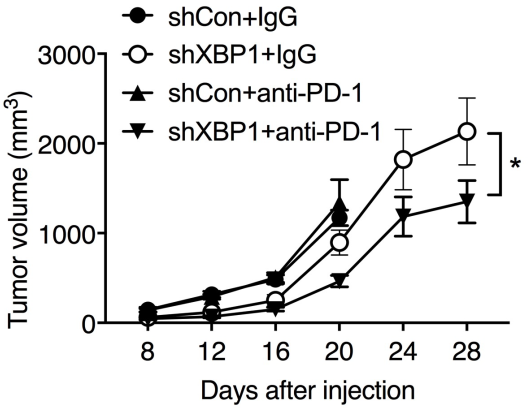

Supplementary Figure 7. Reduced expression of UPR components in murine melanoma responders to anti-CTLA4 treatment. **A**, RT-PCR analysis of indicated UPR-related mRNAs of B2905 melanomas collected from the mice treated with isotype or anti-CTLA4 antibodies (IgG, Non-Responder anti-CTLA4, $n = 5$; Responder anti-CTLA4, $n = 4$). **B**, Tumor growth after s.c. injection of YUMM1.7-shCon and YUMM1.7-shXBP1 cells into C57BL/6 mice treated with control IgG or anti-PD-1 antibody on days 7, 10, 13, and 16 after tumor inoculation ($n = 8$). Data are representative of two independent experiments (**B**) and one experiment (**A**). Graphs show the mean ± s.e.m. *$P < 0.05$, **$P < 0.005$ by one-way ANOVA (**A**) or two-way ANOVA (**B**) with Tukey's correction for multiple comparisons.

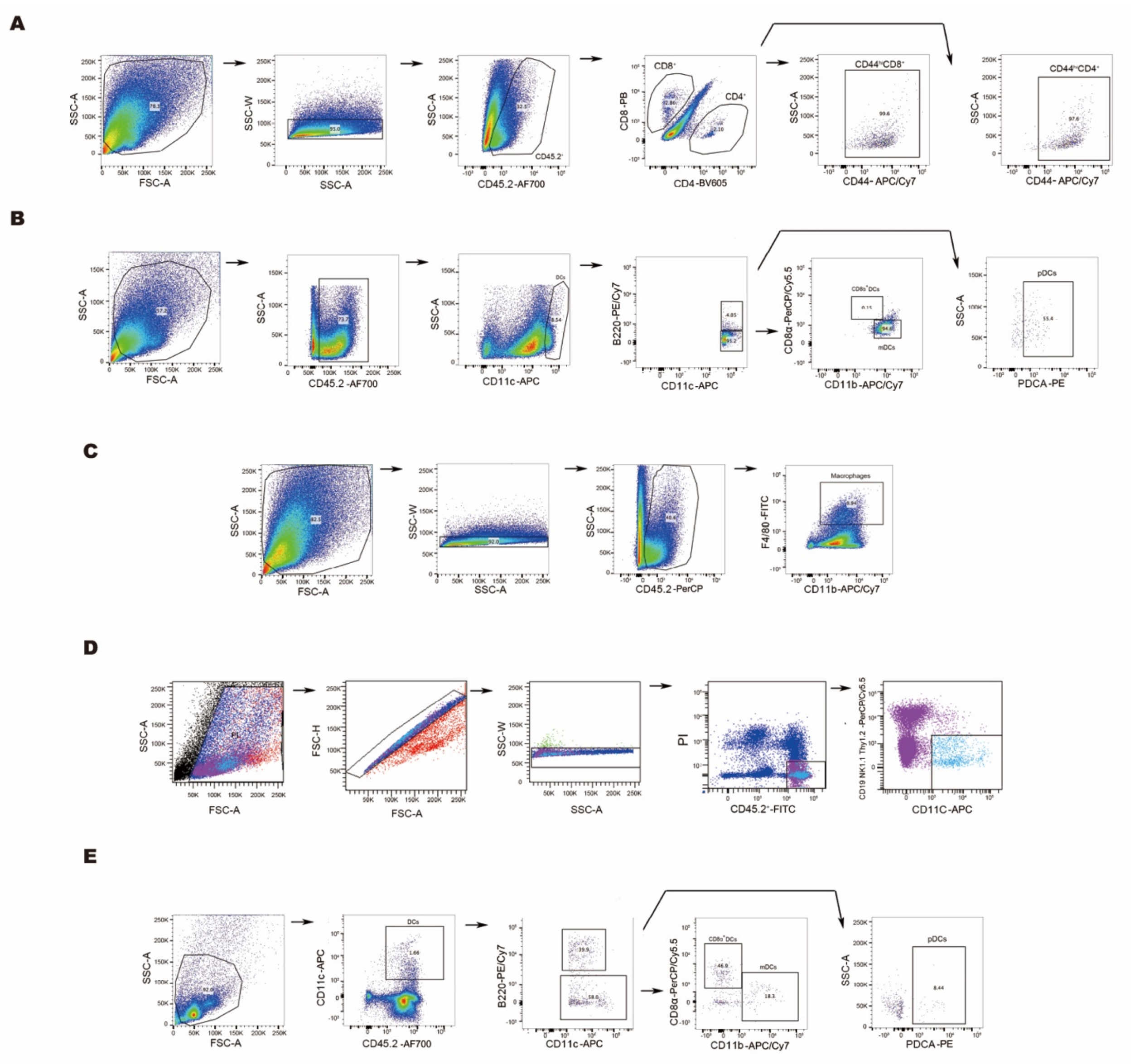

Supplementary Figure 8. Gating strategies for immune cells analysis and cell sorting. **A**, Gating strategy of the tumor infiltrating CD45$^+$, CD44^{high}CD4$^+$ and CD44^{high}CD8$^+$ T cells presented on Figure 1B, 1C, 3E, 4B, 4D, 4F, and Supplementary Figure 1C and 1F. **B**, Gating strategy of the tumor infiltrating DCs (CD45.2$^+$CD11C$^+$), mDCs (CD45.2$^+$CD11C$^+$B220$^-$CD8α$^-$CD11b$^+$), pDCs (CD45.2$^+$CD11C^{int} B220$^+$PDCA$^+$) and CD8α$^+$DCs (CD45.2$^+$CD11C$^+$B220$^-$CD8α$^+$CD11b$^-$) presented on Figure 1D, 1E, 3F and Supplementary Figure 1G, as described before[1]. **C**. Gating strategy of the tumor infiltrating macrophages (CD45$^+$ F4/80$^+$ CD11b$^+$) presented on Supplementary Figure 1E. **D**, Gating strategy of sorted DCs (CD45$^+$CD11C$^+$NK1.1$^-$CD19$^-$Thy1.2$^-$) from PPs presented on Figure 5G, and Supplementary Figure 5G, 5H and 5I. **E**, Gating strategy of the tumor infiltrating DCs, mDCs, pDCs and CD8α$^+$DCs from PPs presented on Figure 5F and Supplementary Figure 5F.

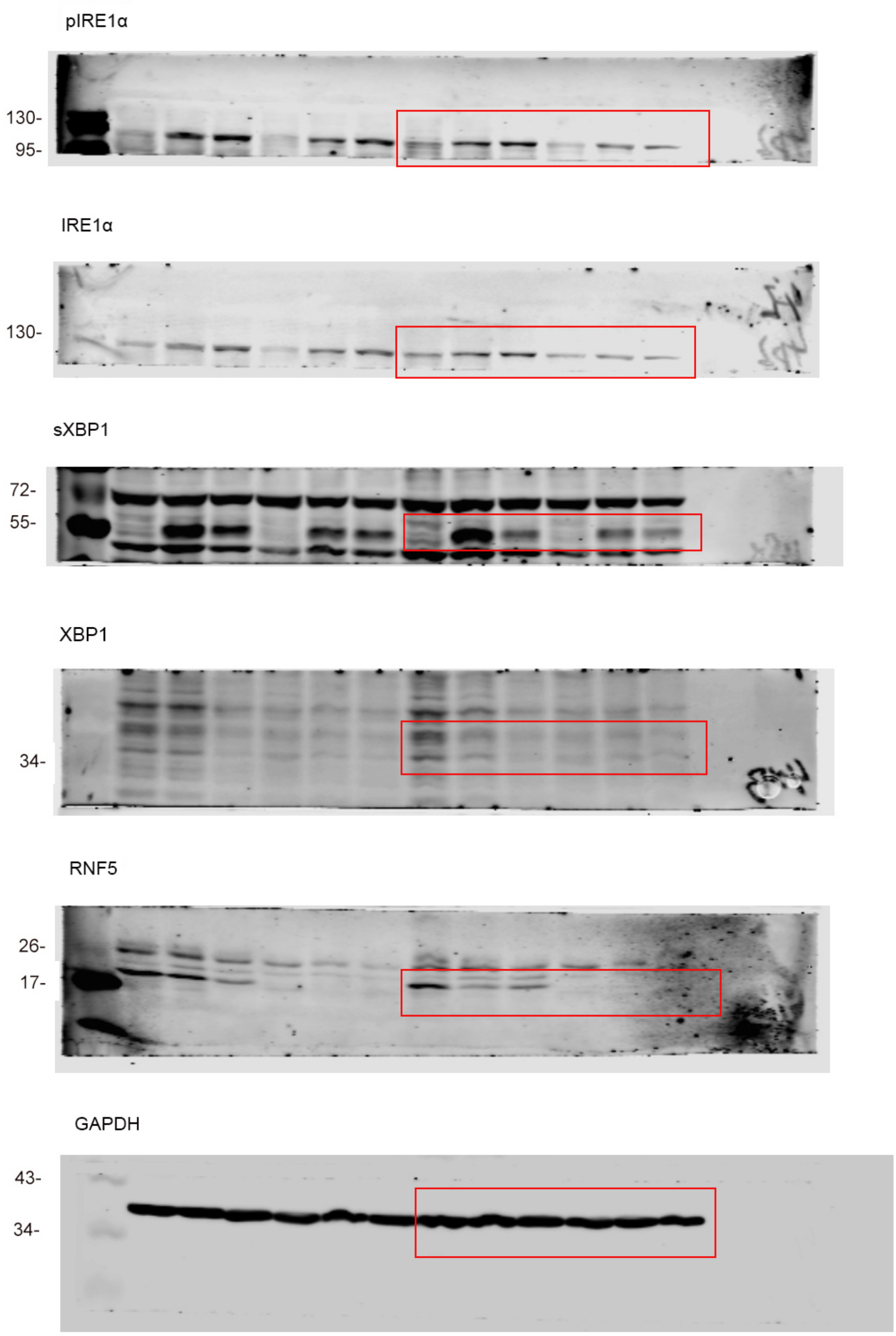

Supplementary Figure 9. raw data for Figure 6A

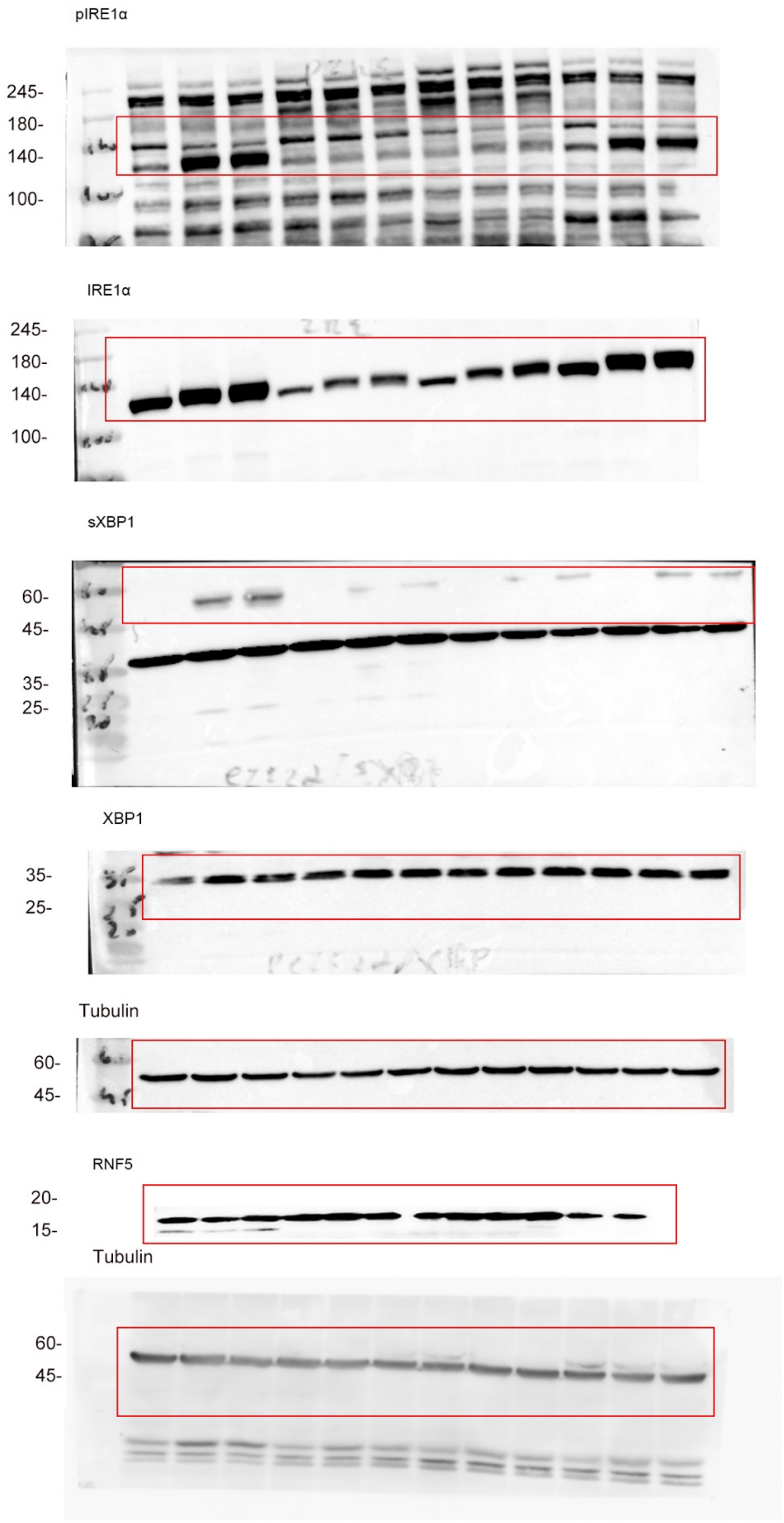

Supplementary Figure 9. raw data for Supplementary Figure 6A

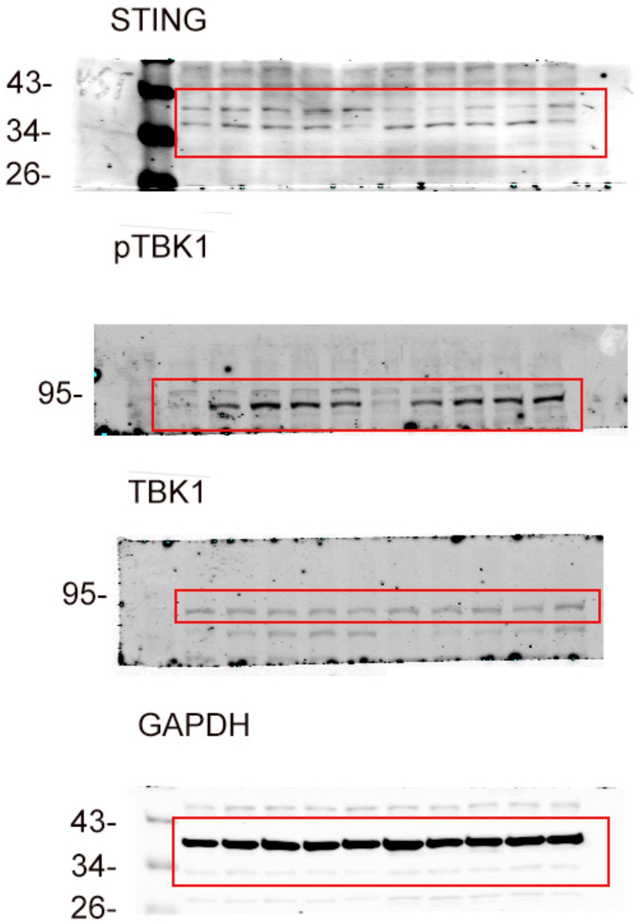

Supplementary Figure 9. raw data for Supplementary Figure 6E

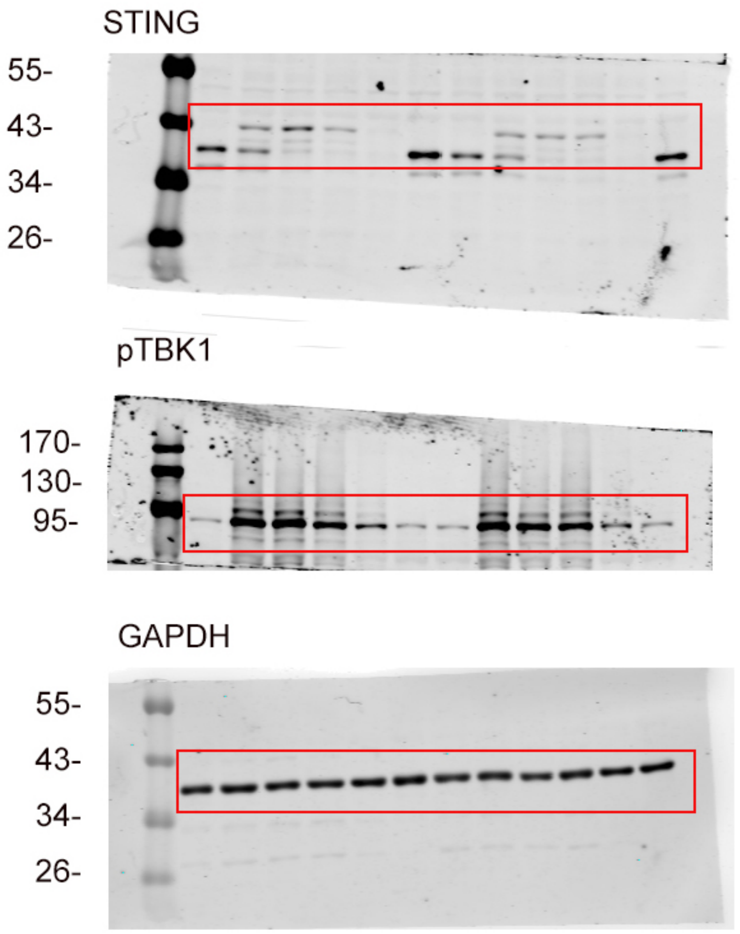

Supplementary Figure 9. raw data for Supplementary Figure 6F

Supplementary Table 1. Bacterial groups that distinguish gut microbiota of WT from *Rnf5*$^{-/-}$ mice. Phylotypes were selected based on abundance and correlation with tumor size at day 24, using Welch *t*-test and Wilcoxon rank-sum test. Benjamini-Hochberg adjusted p-value are less than 0.05.

OSU Group ID	Name	Correlation with tumor volume	Correlation p-value	log10 median of WT day 0	log10 median of WT day 24	log10 median of KO day 0	log10 median of KO day 24	OSU IDs in the group	Mapped Strains
216	Burkholderiales_bacterium/Parasutterella_excrementihominis (92.9-93.9)	-0.51	2.7E-05	-3.41	-3.13	-2.79	-2.49	1009135; 1000742; 1000080	Burkholderiales_bacterium_1_1_47,Parasutterella_excrementihominis_YIT_11859
9	Parabacteroides_sp/Muribaculum_intestinale (92.1-94.1)	-0.49	7.1E-05	-1.14	-0.94	-0.93	-0.79	1004421; 1001550; 1004658; 1007767; 1003515; 1004049; 1009144; 1003893; 1006312; 1002599; 1007466; 1005198; 1003018; 1007913; 1004531; 1003752; 1009332; 1009811; 1008401; 1003032; 1003605; 1004160; 1008447; 1001920; 1001986; 1004579; 1003800; 1001605; 1003821; 1005883; 1005625; 1008098; 1001284; 1007277; 1011959; 1003341; 1007300; 1001166; 1001141; 1010942; 1003513; 1000850; 1003877; 1005710; 1007806; 1005732; 1004093; 1009695; 1009303; 1003027; 1006748; 1002284; 1006800; 1009392; 1002197; 1003310; 1005624; 1005887; 1003833; 1000739; 1003858; 1003724; 1004122; 1003984; 1009015; 1006186; 1004832; 1006644; 1000007; 1000045; 1000172; 1000058; 1005111; 1000362; 1008686; 1001510; 1006971; 1005975; 1009177; 1007410; 1006784; 1005028; 1001006; 1006513; 1008127; 1000167; 1001394; 1004772; 1007584; 1005574; 1005845; 1001365; 1009431; 1002162; 1003346; 1007323; 1004233; 1003666; 1001745; 1002324; 1005954; 1006259; 1005715; 1004070; 1001191; 1005452; 1001427; 1005171; 1005176; 1003260; 1005189; 1004950; 1003013; 1006053; 1005783; 1004135; 1009333; 1007123; 1006804; 1002739; 1001542; 1009005; 1004394; 1002749; 1001998; 1005092; 1002876; 1006250; 1003091; 1001186; 1011385; 1005417; 1006651; 1006000; 1006055; 1005492; 1002356; 1008956; 1009903; 1011682; 1003381; 1009697; 1003016; 1005010; 1006459; 1008538; 1005059; 1000037; 1000146; 1005920; 1009179; 1001448; 1011818; 1003283; 1011508; 1004354; 1001781; 1009907; 1006830; 1004182; 1003110; 1008785; 1004741; 1002727; 1007580; 1007586; 1002455; 1005055; 1001323; 1009862; 1008128; 1003657; 1000891; 1006927; 1005385; 1007328; 1002978; 1003662; 1009141; 1001485; 1004263; 1005143; 1006653; 1006309; 1002166; 1007064; 1001533; 1003417; 1007091; 1004333; 1009337; 1007552; 1006113; 1011166; 1008000; 1006125; 1003474; 1001218; 1002853; 1007262; 1004403; 1010486; 1002765; 1003852; 1006936; 1005945; 1002390; 1005966; 1004072; 1002410; 1002339; 1008287; 1002603; 1007488; 1008359; 1010248; 1006740; 1002362; 1002290; 1006527; 1005096; 1008153; 1007562; 1009905; 1011625; 1009542; 1007048; 1000094; 1007745; 1007435; 1004708; 1007502; 1009369; 1003461; 1005577; 1009445; 1006546; 1006941; 1000497; 1007574; 1010788; 1009511; 1000928; 1010121; 1002209; 1002381; 1003696; 1005446; 1001192; 1002797; 1008810; 1002029; 1008821; 1006072; 1001406; 1002271; 1000902; 1006423; 1010729; 1002829; 1005290; 1002647; 1002741; 1010435; 1004017; 1005095; 1003508; 1004924; 1009247; 1006392; 1011104; 1007525; 1003431; 1003035; 1001829; 1001885; 1004560; 1006453; 1010877; 1007707; 1003861; 1002943; 1004964; 1001198; 1005809; 1006786; 1005264; 1007229; 1008532; 1007244; 1010903; 1008145; 1000018; 1006608; 1000846; 1001064; 1009395; 1007443; 1008055; 1007751; 1008689; 1007439; 1004096; 1003408; 1001104; 1005496; 1001132; 1005513; 1010789; 1003983; 1001784; 1001647; 1007675; 1010489; 1005402; 1003676; 1009184; 1002086; 1005536; 1004145; 1009861; 1001921; 1000975; 1008267; 1007038; 1003920; 1001638; 1002814; 1008824; 1007909; 1002825; 1010325; 1008033; 1000332; 1000338; 1002691; 1004692; 1008799; 1007962; 1000025; 1001857; 1007434; 1001260; 1003674; 1011381; 1003557; 1002808; 1004117; 1002515; 1001381; 1008869; 1010721; 1006436; 1000962; 1008054; 1011254; 1005048; 1002754; 1008615; 1004656; 1003867; 1002344; 1008817; 1002188; 1004552; 1005268; 1009404; 1003198; 1002038; 1005739; 1010280; 1002442; 1000004; 1000019; 1000034; 1001222; 1000648; 1011056; 1001435; 1004942; 1005917; 1001591; 1002494; 1004530; 1007994; 1008026; 1003064; 1009487; 1008203; 1006963; 1003882; 1003905; 1008402; 1004767; 1007045; 1001402; 1002429; 1009856; 1010785; 1011596; 1009460; 1000026; 1005419; 1009554; 1007391; 1002360; 1005682; 1004881; 1009148; 1003002; 1002088; 1010663; 1001322; 1002549; 1011298; 1011671; 1000753; 1000673; 1004702; 1002540; 1003313; 1001503; 1011930; 1009188; 1009830; 1009679; 1001532; 1004249; 1001134; 1011565; 1008580; 1005338	Parabacteroides_sp._YL27,Muribaculum_intestinale_YL27
387	Clostridium_aldenense (93.3-95.3)	-0.44	3.9E-04	-3.27	-4.27	-2.62	-3.08	1008904; 1002719; 1000140	Clostridium_aldenense_RMA_9741
259	Acetatifactor_muris/Lachnoclostridium_sp (93.9-94.8)	-0.44	4.0E-04	-2.65	-2.93	-2.36	-2.49	1005946; 1000068	Lachnoclostridium_sp._An196,Acetatifactor_muris,Acetatifactor_muris_CT-m2
644	Alistipes_senegalensis (94.8-95.7)	-0.43	5.4E-04	-5.13	-5.13	-3.08	-3.32	1005340; 1003482; 1000382; 1000393; 1007761; 1009052	Alistipes_senegalensis_JC50
25	Bacteroides_rodentium/oleiciplenus (94.8-96.5)	-0.40	1.4E-03	-2.09	-2.34	-1.56	-1.80	1009749; 1009283; 1008819; 1000248; 1000727; 1000847; 1000531; 1002183; 1000012	Bacteroides_oleiciplenus_YIT_12058,Bacteroides_oleiciplenus_JCM_16102,Bacteroides_rodentium_JCM_16496
250	Bacteroides_sartorii (100)	-0.35	6.9E-03	-5.12	-3.61	-3.29	-3.21	7357	Bacteroides_sartorii_JCM_17136

622	Clostridium_methylpentosum (91.5-93)	-0.34	8.6E-03	-5.13	-5.12	-3.43	-3.30	1001716; 1005298; 1000813; 1001067; 1011691; 1000355	Clostridium_methylpentosum_DSM_5476,Clostridium_methylpentosum_R2
275	Clostridium_viride (96.5-97.3)	-0.33	9.2E-03	-3.36	-3.16	-3.11	-2.95	1000526; 1000236	Clostridium_viride_DSM_6836
105	Bacteroides_chinchillae/sartorii (100)	-0.33	9.6E-03	-3.20	-3.24	-2.89	-2.90	7358	Bacteroides_chinchillae_JCM_16497,Bacteroides_sartorii_A-C2-0,Bacteroides_sartorii_JCM_16497
70	Bacteroides_chinchillae/sartori (99.8)	-0.33	1.0E-02	-2.97	-2.96	-2.62	-2.56	1000097	Bacteroides_sartorii_dnLKV3,Bacteroides_chinchillae_JCM_16497, Bacteroides_sartorii_A-C2-0,Bacteroides_sartorii_JCM_16497
690	Eubacterium_ventriosum (98)	0.32	1.4E-02	-3.21	-3.27	-5.14	-5.13	1000246	Eubacterium_ventriosum_ATCC_27560
451	Clostridium_sp._ASF356 (93-94.8)	0.33	1.0E-02	-3.04	-4.36	-5.14	-5.15	1000887; 1003995; 1004670; 1004357; 1001462; 1001939; 1005714; 1001326; 1000562; 1000842; 1000702; 1000654; 1000660; 1000508; 1000649; 1001463; 1001736; 1001717; 1004701; 1000911; 1005297; 1001347; 1001328; 1001565; 1000942; 1003772	Clostridium_sp._ASF356
314	Acetatifactor_muri (95.5-97.4)	0.35	6.7E-03	-2.42	-2.68	-2.80	-3.08	1008629; 1002103; 1006121; 1000764; 1010102; 1007804; 1006867; 1001554; 1007453; 1001935; 1006010; 1005964; 1001117; 1011087; 1001433; 1005638; 1000198; 1008313; 1009549; 1002851; 1002335; 1002604; 1004018; 1000351; 1000596; 1002680; 1004900; 1006785; 1000194; 1001179; 1003234	Acetatifactor_muris,Acetatifactor_muris_CT-m2
287	Bifidobacterium_pseudolongum/Actinobacteria_bacterium (100)	0.35	5.5E-03	-4.45	-3.76	-5.14	-5.15	9726	Bifidobacterium_pseudolongum_PV8-2,Actinobacteria_bacterium_UC5.1-1B11,Bifidobacterium_pseudolongum_globosum_1549B,Bifidobacterium_pseudolongum_globosum_1747B,Bifidobacterium_pseudolongum_globosum_1734B
429	Roseburia_sp._499 (96-97)	0.36	4.5E-03	-2.94	-3.12	-3.15	-3.69	1000437; 1008893; 1000231	Roseburia_sp._499
291	Lachnospiraceae_bacterium_TWA4 (95.1-96)	0.37	4.0E-03	-2.51	-2.73	-2.70	-3.33	1000505; 1010679; 1000453; 1002233; 1006654; 1000201; 1000221; 1000409; 1001291; 1000866	Lachnospiraceae_bacterium_TWA4
530	Erysipelothrix_larvae (83.1-84.5)	0.38	2.9E-03	-3.70	-3.54	-5.14	-5.14	1010694; 1011540; 1002468; 1000408; 1011283; 1000627	Erysipelothrix_larvae_LV19
435	Anaerotruncus_colihominis/Clostridiales_bacterium (91.5-92.7)	0.38	2.8E-03	-3.46	-3.56	-5.14	-5.15	1000560; 1005372; 1000521; 1001793; 1011867	Anaerotruncus_colihominis_DSM_17241,Clostridiales_bacterium_VE202-13,Anaerotruncus_colihominis_2789STDY5834939,Anaerotruncus_colihominis_An251,Anaerotruncus_colihominis_An175,Anaerotruncus_colihominis_An174,Anaerotruncus_colihominis_WAL_14565
233	Lachnospiraceae_bacterium_A2 (95.3-97.2)	0.38	2.7E-03	-2.45	-2.56	-2.66	-2.93	1010501; 1002399; 1002708; 1003024; 1007329; 1000092; 1007401; 1000325; 1000668; 1002879; 1004035; 1005670; 1006234; 1006628; 1004677; 1003081; 1005500; 1010741; 1011419; 1005469	Lachnospiraceae_bacterium_A2
266	Lachnospiraceae_bacterium/Roseburia_sp (93.5)	0.38	2.6E-03	-3.25	-3.31	-5.14	-5.14	1000322	Lachnospiraceae_bacterium_3-1,Roseburia_sp._499
56	Lachnospiraceae_bacterium_A4 (98.4-99.8)	0.38	2.6E-03	-1.92	-2.10	-2.78	-2.59	1003987; 1000033	Lachnospiraceae_bacterium_A4
549	Eggerthella_sp._YY7918 (93)	0.38	2.4E-03	-4.25	-3.97	-5.14	-5.15	1000605	Eggerthella_sp._YY7918
184	Bifidobacterium_pseudolongum (100)	0.39	2.1E-03	-3.29	-3.02	-3.79	-3.76	7625	Bifidobacterium_pseudolongum_UMB-MBP-01
253	Lachnospiraceae_bacterium/Clostridium_sp (96.5-96.8)	0.39	1.9E-03	-1.45	-1.61	-2.33	-2.06	1000020; 1000021	Lachnospiraceae_bacterium_A4, Clostridium_sp._KNHs209
245	Neglecta_sp (97.8)	0.40	1.4E-03	-2.63	-2.68	-3.13	-5.13	1000126	Neglecta_timonensis_SN17,Neglecta_sp._Marseille-P3890
743	Acetatifactor_muris/Clostridium_sp (95.3)	0.41	1.1E-03	-3.95	-3.86	-5.14	-5.15	1000468	Clostridium_sp._KNHs209,Acetatifactor_muris,Acetatifactor_muris_CT-m2
354	Intestinimonas_massiliensis (95.1-96.1)	0.44	4.9E-04	-3.08	-3.19	-4.23	-5.14	1011774; 1000245	Intestinimonas_massiliensis_GD2
155	Eubacterium_plexicaudatum (98.8)	0.44	4.6E-04	-2.63	-2.87	-2.90	-3.13	1000083	Eubacterium_plexicaudatum_ASF492
453	Clostridium_sp._ASF356 (98.3-99.8)	0.45	3.5E-04	-2.68	-2.67	-2.87	-2.93	1000163; 1000376; 1000274; 1000388	Clostridium_sp._ASF356
528	Desulforegula_conservatrix (80.1)	0.47	1.3E-04	-3.81	-3.77	-5.14	-5.15	1000476; 1009965	Desulforegula_conservatrix_Mb1Pa

116	Clostridium_populeti (93.6-95.5)	0.48	1.1E-04	-2.56	-1.92	-3.77	-5.13	1007464; 1008552; 1002159; 1000968; 1000096; 1010727; 1003066; 1000098; 1007217; 1001569; 1000564; 1003779; 1000055; 1000438; 1002461; 1002670; 1004514; 1000446; 1003172; 1001893; 1000509; 1000428	Clostridium_populeti_743A
153	Eubacterium_plexicaudatum (93.2-95.1)	0.50	5.2E-05	-1.93	-1.97	-2.74	-2.52	1004104; 1000029; 1000209; 1000378; 1000903; 1002068; 1008650; 1003766; 1003967; 1006687; 1007582; 1003350; 1009582; 1001283; 1002382; 1004060; 1001070; 1002919; 1002962; 1003887; 1005818; 1001077; 1004334; 1005821; 1004681; 1007313; 1006597; 1005007; 1005599; 1004385; 1004391; 1006523; 1004899; 1003569; 1004779; 1003791; 1001860; 1002089; 1002191; 1010826; 1001302; 1003570; 1008811; 1001053; 1003705; 1003055; 1006356; 1003794; 1000985; 1001164; 1000176; 1002675; 1006311; 1001089; 1003727; 1002522; 1007531; 1003617; 1001932; 1003358; 1000856; 1005846; 1008928; 1001631; 1001208; 1007092; 1001538; 1008605; 1008178; 1000916; 1000920	Eubacterium_plexicaudatum_ASF 492
308	Lachnospiraceae_bacterium/Dorea_sp (98.8-99)	0.51	3.1E-05	-2.92	-2.89	-5.13	-5.14	1000419; 1000218	Dorea_sp._5-2,Lachnospiraceae_bacterium_3-2
117	Clostridium_populeti (95.8-96.5)	0.52	1.8E-05	-3.00	-2.47	-3.44	-3.83	1000498; 1000101; 1000405; 1002850; 1000931	Clostridium_populeti_743A
224	Eubacterium_ramulus (94.6-96.5)	0.57	2.5E-06	-2.56	-2.61	-3.19	-3.39	1005026; 1000102	Eubacterium_ramulus_ATCC_29099,Eubacterium_ramulus_2789STDY5608891
480	Clostridium_xylanolyticum/Bacteroides_xylanolyticus (95.3-96.3)	0.58	1.3E-06	-2.76	-3.01	-5.13	-5.14	1010154; 1000193	Clostridium_sp._12(A),Clostridium_xylanolyticum_DSM_6555,Bacteroides_xylanolyticus_X5-1
436	Anaerotruncus_colihominis/Clostridiales_bacterium (93.8-95.8)	0.58	1.1E-06	-2.95	-2.77	-3.18	-3.38	1001009; 1008169; 1000243; 1000251	Anaerotruncus_colihominis_DSM_17241,Clostridiales_bacterium_VE202-13,Anaerotruncus_colihominis_2789STDY5834939,Anaerotruncus_colihominis_An251,Anaerotruncus_colihominis_An175,Anaerotruncus_colihominis_An174,Anaerotruncus_colihominis_WAL_14565

Supplementary Table 2. Species for the bacterial cocktail were as follows:

**Species name**	**Source**	**Catalog number**
Alistipes finegoldii	DSMZ	17242
Alistipes putredinis	ATCC	29800
Bacteroides massiliensis	DSMZ	17679
Bacteroides rodentium	DSMZ	26882
Bacteroides sartorii	DSMZ	21941
Clostridium clostridioforme	ATCC	25537
Clostridium methylpentosum	ATCC	43829
Lactobacillus animalis	ATCC	35046
Lactobacillus murinus	ATCC	35020
Muribaculum intestinale	DSMZ	28989
Oscillibacter valericigenes	DSMZ	18026
Parasutterella excrementihominis	DSMZ	21040

Supplementary Table 3. Patient characteristics of MGH/Dana-Farber/Harvard Cancer Center (USA) cohort.

Patient	Sex	Age	Response	Therapy	Progression-free Survival (days)	Overall Survival (days)
1	F	45	R	NIVO+aKIR	1317	1317
2	F	63	R	IPI+NIVO	776	776
3	M	74	R	pembro	57	470
4	M	48	NR	IPI	81	1075
5	M	80	NR	NIVO	80	476
6	F	78	R	pembro	555	789
7	F	67	NR	pembro (prior vem+cobi)	58	714
8	M	67	NR	IPI+NIVO	324	599
9	M	70	NR	pembro	74	345
10	M	78	NR	pembro	182	548
11	F	69	NR	pembro	147	895
12	M	65	NR	pembro	81	144
13	M	74	NR	pembro	103	354
14	M	28	R	IPI+NIVO	789	789
15	M	77	NR	pembro	474	783
16	M	65	R	pembro	624	624
17	F	54	R	ipi+nivo+GM-CSF	578	578
18	M	73	R	IPI+NIVO	592	592
19	M	52	R	IPI+NIVO	264	264
20	M	77	R	pembro	571	571
21	M	75	R	pembro	404	404
22	F	58	NR	IPI+NIVO	78	183
23	M	53	R	pembro (adjuvant)	326	326
24	F	70	NR	pembro	103	354
25	F	67	NR	IPI+NIVO	76	313
26	M	75	NR	pembro+epacadostat	212	326
27	M	82	NR	IPI+NIVO	15	15
28	M	71	NR	pembro+entinostat (prior pembro)	74	232
29	F	54	R	pembro (adjuvant)	144	144
30	F	54	R	IPI+NIVO (adjuvant)	140	140
31	F	57	NR	IPI+NIVO	76	187
32	M	70	R	IPI+NIVO	210	210
33	F	70	R	IPI+NIVO	204	204
34	F	69	R	pembro (adjuvant)	74	74
35	M	73	R	IPI+NIVO	89	89
36	M	59	R	IPI+NIVO	109	109
37	F	72	NR	pembro	49	86
38	M	71	R	IPI (prior NIVO)	1071	1391
39	M	61	NR	IPI	32	45
40	M	55	R	aPD1	1861	1861

Supplementary Table 4. Patient characteristics of Zurich Hospital (Switzerland) cohort.

Patient	Sex	Age	Response to Ipilimumab
1	F	47	R
2	M	77	NR
3	M	54	NR
4	M	72	R
5	F	44	R
6	M	72	NR
7	M	48	NR
8	M	56	NR
9	F	54	NR
10	M	69	R
11	M	57	R
12	F	47	R
13	F	66	NR
14	M	34	NR
15	M	73	NR
16	F	36	R
17	F	67	NR
18	F	65	NR
19	M	59	R
20	F	59	NR
21	F	73	NR
22	M	46	NR
23	M	51	NR
24	F	69	R
25	F	54	NR

Supplementary Table 5. Patient characteristics of Rambam Health Care Center (Israel) cohort.

Patient	Sex	Year of Birth	Response to Nivolumab/Pembrolizumab	Disease-free Survival(months)	Overall Survival(months)
1	M	1958	NR	4	9
2	M	1941	R	23	28
3	M	1931	R	21	21
4	F	1956	R	13	21
5	M	1963	R	28	28
6	M	1931	NR	8	10
7	M	1934	R	12	12
8	M	1922	NR	3	4
9	M	1942	NR	4	8
10	F	1951	NR	6	13
11	M	1938	R	19	19
12	M	1940	R	39	39
13	F	1950	R	29	29
14	F	1963	R	23	23
15	F	1953	R	35	37
16	F	1939	R	36	36
17	F	1951	NR	4	16
18	M	1947	NR	3	5
19	M	1963	NR	3	8
20	M	1936	NR	5	25
21	M	1953	NR	2	7

Supplementary Table 6. Primers for qRT-PCR analyses were as follows

**Gene**	**Forward (5'-3')**	**Reverse (5'-3')**
**Mouse**		
CCL2	TTAAAAACCTGGATCGGAACCAA	GCATTAGCTTCAGATTTACGGGT
CCL4	TTCCTGCTGTTTCTCTTACACCT	CTGTCTGCCTCTTTTGGTCAG
CCL5	GCTGCTTTGCCTACCTCTCC	TCGAGTGACAAACACGACTGC
CCL8	TCTACGCAGTGCTTCTTTGCC	AAGGGGGATCTTCAGCTTTAGTA
TLR4	ATGGCATGGCTTACACCACC	GAGGCCAATTTTGTCTCCACA
TLR7	ATGTGGACACGGAAGAGACAA	GGTAAGGGTAAGATTGGTGGTG
TLR9	ATGGTTCTCCGTCGAAGGACT	GAGGCTTCAGCTCACAGGG
CD80	ACCCCCAACATAACTGAGTCT	TTCCAACCAAGAGAAGCGAGG
CD86	TGTTTCCGTGGAGACGCAAG	TTGAGCCTTTGTAAATGGGCA
CD40	GCTGTGAGGATAAGAACTTGGAG	CTGGTTCGACAGGGCTGAA
Stat1	CGGAGTCGGAGGCCCTAAT	ACAGCAGGTGCTTCTTAATGAG
ICOS1	ATGAAGCCGTACTTCTGCCG	CGCATTTTTAACTGCTGGACAG
KLRC1	GCCCCTGCAAAGGTTTTCC	TCTGTGGGTTCTAGTCATTGAGG
KLRD1	TCTAGGATCACTCGGTGGAGA	CACTTGTCCAGGCAAACACAG
sXBP1	CTGAGTCCGAATCAGGTGCAG	GTCCATGGGAAGATGTTCTGG
IFN-α	TGCCCAGCAGATCAAGAAGG	TCAGGGGAAATTCCTGCACC
IL-1β	GCAACTGTTCCTGAACTCAACT	ATCTTTTGGGGTCCGTCAACT
TNF-α	CCCTCACACTCAGATCATCTTCT	GCTACGACGTGGGCTACAG
IL-6	TAGTCCTTCCTACCCCAATTTCC	TTGGTCCTTAGCCACTCCTTC
NLRP3	ATTACCCGCCCGAGAAAGG	TCGCAGCAAAGATCCACACAG
NLRP6	CTCGCTTGCTAGTGACTACAC	AGTGCAAACAGCGTCTCGTT
NOD2	CAGGTCTCCGAGAGGGTACTG	GCTACGGATGAGCCAAATGAAG
XBP1	AGCAGCAAGTGGTGGAATTG	GAGTTTTCTCCCGTAAAAGCTGA
ERdj4	CTCCACAGTCAGTTTTCGTCTT	GGCCTTTTTGATTTGTCGCTC
Agpat6	AGCTTGATTGTCAACCTCCTG	CCGTTGGTGTAGGGCTTGT
Fasn	GGAGGTGGTGATAGCCGGTAT	TGGGTAATCCATAGAGCCCAG
SCD2	GCATTTGGGAGCCTTGTACG	AGCCGTGCCTTGTATGTTCTG
Lpar1	GCTTCTACAATGAGTCTATCGCC	TGATGAACACGCAAACAGTGAT
CHOP	CTGGAAGCCTGGTATGAGGAT	CAGGGTCAAGAGTAGTGAAGGT
ATF4	CCTGAACAGCGAAGTGTTGG	TGGAGAACCCATGAGGTTTCAA
MMP7	CTGCCACTGTCCCAGGAA	GGGAGAGTTTTCCAGTCATGG
Phospholipases A2	CAGCACATTATAGTGGAACACCA	AGTGTCCAGCATATCGCCAAA
Angiogenin-4	GGTTGTGATTCCTCCAACTCTG	CTGAAGTTTTCTCCATAAGGGCT
RNF5	AAGAATGCCCGGTGTGTAAAG	GCGGGGTGGAGTTTTCAATC
18S	GTAACCCGTTGAACCCCATT	CCATCCAATCGGTAGTAGCG
Tubb5	GATCGGTGCTAAGTTCTGGGA	AGGGACATACTTGCCACCTGT
**Human**		
sXBP1	CCGCAGCAGGTGCAGG	GAGTCAATACCGCCAGAATCCA

BIP	CATCACGCCGTCCTATGTCG	CGTCAAAGACCGTGTTCTCG
ATF4	CCCTTCACCTTCTTACAACCTC	TGCCCAGCTCTAAACTAAAGGA
**Bacteria**		
Bacteroides	GAGAGGAAGGTCCCCCAC	CGCTACTTGGCTGGTTCAG
Lactobacillus	GAGGCAGCAGTAGGGAATCTTC	GGCCAGTTACTACCTCTATCCTTCTTC
Alistipes finegoldii	GTACTAATTCCCCATAACATTCGAG	CTAATACAACGCATGCCCATCTT
V3-V4	CCTACGGGNGGCWGCAG	GACTACHVGGGTATCTAATCC
Bacteroides massiliensis	CAAGGCCTACAAACGCAGTG	GCGGATGGTCCTCTTGGG

Supplementary References

1. Fuertes, M.B., *et al.* Host type I IFN signals are required for antitumor CD8+ T cell responses through CD8{alpha}+ dendritic cells. *J Exp Med* **208**, 2005-2016 (2011).
